# Molecular exclusion limits for diffusion across a porous capsid

**DOI:** 10.1038/s41467-021-23200-1

**Published:** 2021-05-18

**Authors:** Ekaterina Selivanovitch, Benjamin LaFrance, Trevor Douglas

**Affiliations:** 1 Department of Chemistry, Indiana University, Bloomington, IN USA; 2 Department of Molecular and Cell Biology, University of California Berkeley, Berkeley, CA USA

**Keywords:** Nanobiotechnology, Biomaterials, Nanoparticles

## Abstract

Molecular communication across physical barriers requires pores to connect the environments on either side and discriminate between the diffusants. Here we use porous virus-like particles (VLPs) derived from bacteriophage P22 to investigate the range of molecule sizes able to gain access to its interior. Although there are cryo-EM models of the VLP, they may not accurately depict the parameters of the molecules able to pass across the pores due to the dynamic nature of the P22 particles in the solution. After encapsulating the enzyme AdhD within the P22 VLPs, we use a redox reaction involving PAMAM dendrimer modified NADH/NAD+ to examine the size and charge limitations of molecules entering P22. Utilizing the three different accessible morphologies of the P22 particles, we determine the effective pore sizes of each and demonstrate that negatively charged substrates diffuse across more readily when compared to those that are neutral, despite the negatively charge exterior of the particles.

## Abstract (web summary)

Molecular transport across physical barriers requires pores that connect to the environment. Here, the authors report on a solution based dynamic study into the effects of size and charge on the transport through the pores of virus-like particles derived from bacteriophage P22 using an encapsulated redox system.

## Introduction

Physical barriers, and the molecular communication across those barriers, are fundamental themes in biology^1–3^. Among other features, biological barriers provide cellular and sub-cellular separation with controlled permeability. The diffusion of molecules across barriers is often selective and is usually made possible via channels or pores that can discriminate based on molecular properties such as size, charge, and chemical makeup^4–8^. Pores, gates, and channels are often responsible for homeostasis by controlling chemical fluctuations across the barrier. Porous barriers are not exclusive to biological systems and they are used extensively in many materials fields such as catalysis, energy, gas storage, semi-conductors, purification, and separations, among others^9–12^. The advantages of porous materials often align with the advantages provided by pores in biological systems.

A eukaryotic cell provides many sophisticated examples of porous barriers in the form of membranes. The cell itself has a defining semi-permeable membrane and internally contains many membrane-bound organelles where diffusion across is controlled via channels. An example is the nucleus, where the nuclear pore complex limits the diffusion of molecules smaller than 9 nm in either direction^13^. Bacterial cells are also defined by semi-permeable membranes, but sub-cellular bacterial microcompartments, such as the carboxysome, are assembled from proteins and package enzymes that facilitate specialized metabolic reactions. The carboxysome is a proteinaceous compartment where the porous shell acts as a molecular barrier and is thought to discriminate between O_2_ and CO_2_ by selectively limiting diffusion of O_2_ through the pores^14–16^. While channels and pores are key features that serve as communications portals across the barriers^17–19^, the mechanisms of diffusion across biological pores remains a major topic of interest.

Viruses and virus-like particles (VLPs) provide additional examples of porous biological systems. Many viral capsids comprise porous protein shells whose function is to protect their encapsulated genome while allowing the diffusion of water and ions across the pores^20^. The capsids of VLPs are structurally similar to the viruses from which they are derived but are non-infectious and can be repurposed to sequester a variety of different cargos including proteins, enzymes, small molecules, polymers, and even other protein compartments^21–25^. For example, VLPs derived from the *Salmonella typhimurium* virus bacteriophage P22 serve as versatile porous protein cages that can be repurposed to encapsulate a diverse array of cargo molecules. P22 VLPs self-assemble into 56 nm *T* = 7 icosahedral capsids from 420 units of a coat protein (CP) and ~100–300 copies of a scaffolding protein (SP). The SP templates assembly of P22 and, upon formation of the particles, is encapsulated as a cargo^26–30^. Genetically fusing other proteins to the SP has allowed for the encapsulation of cargo molecules in high copy-number, with local concentrations mimicking that of the intracellular environment, while maintaining the native morphology of the P22 particles^31–35^.

P22 VLPs provide a unique system to study the molecular selectivity for diffusion across a porous barrier connecting the interior of a compartment to the bulk environment. These particles offer the advantage of the controlled encapsulation of enzymes and provide access to three different particle morphologies, each with a different pore size. The first of these morphologies is the procapsid (PC). In both the infectious phage and the VLP system, PC is the initial structure formed by SP templated assembly and has ~3 nm pores located at the center of the hexameric CP units^26,30^. In the infectious phage, a morphological transformation takes places during DNA packaging resulting in an expansion of the PC structure to a more angular particle 62 nm in diameter but with smaller pores^36,37^. This morphogenesis can also be induced in VLPs with either heat or chemical treatment and the resulting particles are referred to as the expanded morphology (EX)^27,38,39^. The third morphology, known as the wiffle-ball (WB), is accessed by heating the VLP capsids at 75 °C^39^. The diameter of WB is the same as EX, but the loss of all 12 of the pentameric units in the icosahedral capsid creates extremely large 10 nm pores. While there are static approximations of these three structures (PC, EX, and WB) and some information available on the modeled dynamics of other capsids in an aqueous environment^40,41^, the dynamics and effective porosity of P22 are not resolved.

**Fig. 1:**
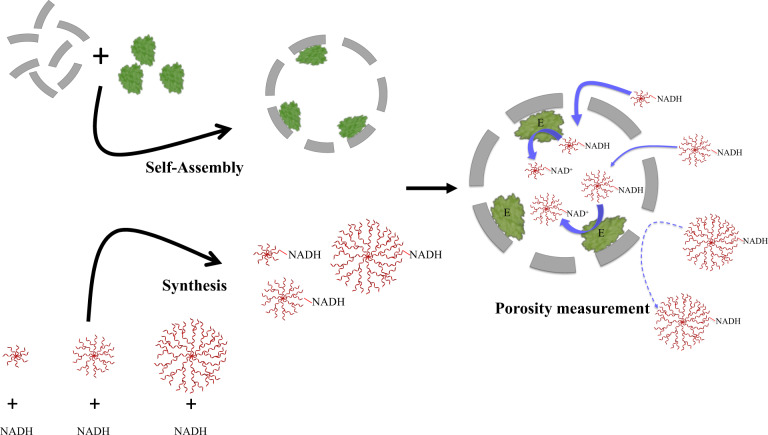
Scheme of our experiments. P22 particles self-assembled with coat protein (CP = gray) and scaffold protein (SP)-enzyme fusion (AdhD-SP = green). NADH was synthesized to yield NADH-dendrimer conjugates (red). The porosity of P22 particles was probed by using the various NADH-dendrimer molecules as bait for the AdhD enzyme. If the “bait” accessed the interior of the particle, we saw the redox reaction (NADH→NAD^+^).

In this study, we investigate the porosity of P22 VLPS and develop a methodology to study diffusion across porous barriers. Since barriers have been known to contribute to the selectivity of enzymes, we also determine how diffusion across the P22 pores contributes to this. We use the activity of an encapsulated enzyme to probe the access of different synthetic substrates across the porous barrier of the P22 VLP. By introducing substrates of different sizes, we can experimentally determine how the size-dependent porosity of the P22 particle limited molecular access to the encapsulated enzymes (Fig. 1). The three different P22 morphologies each exhibit different gating selectivity thresholds due to their different pore sizes. We demonstrate that although the biochemically determined effective pore sizes for P22 particles are comparable to the pore size calculated from the available cryo-EM structures, after the side chain electron density is taken into account the effective pore sizes appear much larger than the P22 model, suggesting that the capsid might indeed be dynamic and undergo a “breathing” mechanism. Furthermore, an examination of the role of substrate charge reveals that negatively charged substrates were capable of traversing across the P22 barrier more readily than the neutral substrates. Although the exterior of the P22 VLPs exhibits a net negative charge, the electrostatic environment immediately surrounding the pores shows that there are both negatively and positively charged regions that can account for differences in charge selectivity. The importance of electrostatic effects on diffusion becomes even more pronounced upon alteration of the P22 charge distribution through the construction of a series of mutants. We see that after removing the positive regions found around the pore interior, the diffusion of substrates across the pore is inhibited. Overall, this study provides a generalizable approach to probe the effective permeability of artificial nano-reactors and contributes to fundamental insights into how compartments regulate substrate diffusion.

## Results and discussion

### Characterization of P22 VLPs

**Fig. 2:**
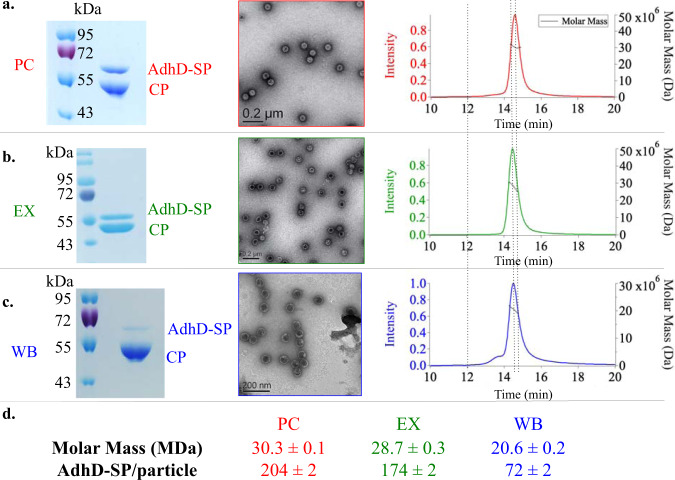
Characterization of P22 VLPs. SDS-PAGE, TEM, and SEC-MALS analysis of **a** procapsid (PC = red), **b** expanded (EX = green), and **c** wiffle-ball (WB = blue) morphologies. **d** Molar mass as determined by SEC-MALS and average number of AdhD-SP cargo molecules per P22 particle. These are representative images and chromatograms of at least 5 independent experiments for the SDS-PAGE, 20 TEM micrographs, and at least 3 for the SEC-MALS.

P22 VLPs were prepared with encapsulated alcohol dehydrogenase-D (AdhD) enzymes from *Pyrococcus furiosus*, using previously established methodologies^32^. Expression of CP and SP-AdhD fusion resulted in the assembly of PC VLPs. The PC particles were characterized using denaturing sodium dodecyl sulfate polyacrylamide gel electrophoresis (SDS-PAGE), transmission electron microscopy (TEM), and size-exclusion chromatography coupled with multi-angle light scattering (SEC-MALS) (Fig. 2a). SDS-PAGE analysis revealed the presence of two bands with molecular weights consistent with CP (46.7 kDa) and the AdhD-SP (52.2 kDa). TEM images showed fully formed 56 nm spherical particles, while SEC-MALS showed a single main peak in the elution profile and determination of the molar mass of the particles allowed us to calculate the average number of cargo AdhD enzymes encapsulated (~204/capsid). The average cargo number provided by SEC-MALS was in agreement with the relative band ratios on the SDS-PAGE gel. Morphological transformation of PC to EX structure was accomplished by treatment with SDS and resulted in complete EX formation (Fig. 2b). SDS-PAGE showed the presence of two bands with a slightly reduced AdhD-SP band intensity^27,38^. TEM displayed slightly more angular particles with a 62 nm diameter, consistent with the expected 10% size increase resulting from the PC→EX morphological transformation. Analysis by SEC-MALS of this sample showed a single main peak in the chromatogram at shorter retention time consistent with an increase in particle size of the EX. From the molar mass we determined an average loading of 174 AdhD-SP/VLP. This slight decrease in average number of cargo molecules is likely due to disruption of particles with the highest density of cargo molecules upon exposure to SDS^38^. The formation of the other morphological form of P22, WB, was accomplished by heating PC sample to 75 °C (Fig. 2c). Although the pores of the WB capsid are theoretically big enough for the enzyme cargo to escape, it has previously been shown that a small fraction of cargo molecules remain encapsulated perhaps due to non-specific interactions between AdhD within the capsid or AdhD aggregation within the capsid^32,35^. SDS-PAGE analysis of purified WB samples confirmed a band at the expected MW for SP-AdhD, indicating the retention of ~70 enzymes within the WB VLPs. WB particles imaged by TEM were spherical with 62 nm diameters and showed gaps in the electron densities of the capsid shell presumably from the sites of the missing pentamers, typical for WB particles. The peak on the SEC-MALS chromatogram showed the same retention time as the EX chromatogram above and analysis of the particle mass provided an average of 72 AdhD-SP/particle. The average number of AdhD-SP in this sample might have been slightly over-estimated due to the molar mass contributions from particles that have undergone an incomplete transformation and not all pentamers have dissociated from the capsid. For our study, this slight overestimation is negligible because we monitored relative changes in enzyme activity. All three of the morphologies (PC, EX, WB) were compared on a native agarose gel to evaluate their relative electrophoretic mobility (Supplementary Fig. 1). Each lane showed a single band, suggesting a single species present in each sample, with characteristic migration for the expected morphology.

### Characterization and NADH of PAMAM dendrimers-NADH conjugates

To establish a range of substrate sizes with which to probe the P22 VLP porosity, the NADH cofactor for the AdhD enzyme was conjugated to different sized PAMAM dendrimers. Six different sizes (1.3, 2.5, 3.4, 4.3, 6.4, and 7.8 nm diameter) of NADH-Dendrimer conjugates were synthesized for this work, using slightly adjusted conditions from established protocols for nicotinamide adenine dinucleotide (NAD) modification^42–46^. The overall schematic of the NAD-dendrimer synthesis is shown in Supplementary Fig. 2 and detailed synthesis can be found in the Methods section. The modified NADH was conjugated to polyamidoamine (PAMAM) dendrimers of increasing generations (Gen 0.5–5.5), followed by characterization using NMR, dynamic light scattering (DLS), and zeta potential measurements. NADH-NH_2_ was used as the starting material for conjugation to carboxylate-terminated PAMAM dendrimers, using standard EDC coupling. All NADH conjugates (NADH-Neg) were analyzed by DLS and zeta potential (Table 1 and Supplementary Figs. 8 and 9). DLS revealed an approximately 1 nm increase in the hydrodynamic radius (*R*_*H*_) when comparing stock dendrimers Gen 0.5–Gen 5.5 to those that were coupled with NADH-NH_2_, likely due to the addition of NADH molecules to the dendrimers. The theoretical dimensions of NADH-NH_2_ are approximately 1.3 nm × 2.2 nm in the extended conformation and are shown in Supplementary Fig. 10. Zeta potential remained negative for all generations albeit slightly less negative for the modified dendrimers, likely due to the decrease in the number of carboxylate groups upon addition of NADH-NH_2_. As the generations increased, the difference in zeta potential also increased with the biggest difference being between NADH-Neg_0.5_ and NADH-Neg_5.5_; −25 and −18 mV respectively. Neither zeta potential nor DLS data are shown for Gen 0.5 due to the small size of the molecule that results in large error in these measurements. ^1^H NMR  data were also obtained for the modified dendrimers (Supplementary Figs. 11–16) and the number of NADH-NH_2_ molecules per dendrimer was calculated for all generations using protons that were adjacent to the amide nearest the terminal carboxylic acid (Supplementary Fig. 11), identified in previous studies of PAMAM dendrimers^47^. The C2 proton of the adenine was also identified; however, as the generations increased, the signal-to-noise ratio was too low for quantitative analysis, though qualitatively the downfield C2 proton was still observed. The coupling reaction was also verified using UV spectroscopy on purified materials, where the peak at 340 nm indicated the presence of the conjugated NADH molecules.

**Table 1:** Details on stock dendrimers and values used to calculate molar ratios for synthesis and measured *R*_*H*_ values of NADH-Neg dendrimers.

Dendrimer generation	Gen 0.5	Gen 1.5	Gen 2.5	Gen 3.5	Gen 4.5	Gen 5.5
Theoretical MW (g/mol)	1269	2935	6267	12,931	26,258	52,913
No. of terminal groups	8	16	32	64	128	256
Molar ratio NADH:Dendrimer	1	1	2	4	8	16
*R*_*H*_ (nm)	1.26 ± 0.20	2.53 ± 0.3	3.39 ± 0.06	4.33 ± 0.02	6.44 ± 0.06	7.79 ± 0.06

### Size discrimination of substrates diffusing across the P22 capsid shell

Probing the porosity of P22 VLPs revealed that there is a discrete pore size that limits chemical communication and discriminates between differently sized molecules traversing across the capsid. This allowed us to calculate the effective pore size for the different morphological forms of P22 VLPs. The estimated pores sizes were in good agreement with the pore sizes calculated from the available cryo-EM reconstruction models but differed once we accounted for the steric contributions from the amino acid side chains, suggesting that the flexible regions of the pores may undergo some size fluctuations.

**Fig. 3:**
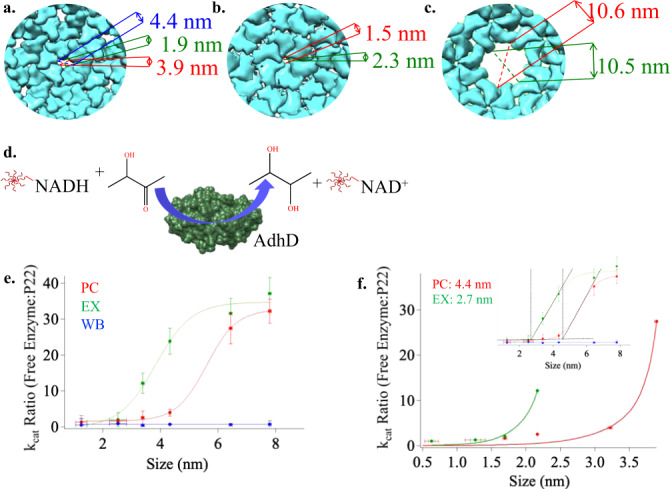
Pore sizes based on available CRYO-EM data and measured effective pores sizes of P22. **a**–**c** Estimated pore dimensions of procapsid (PC) (**a**), expanded (EX) (**b**), and wiffle-ball (WB) (**c**) using Chimera software program. **d** Reduction of acetoin using NADH conjugate as a cofactor and AdhD as a catalyst. **e** Plot showing *k*_cat_ ratio using enzymes AdhD-SP (free) to encapsulated in PC (red), EX (green), and WB (blue) . **f** Aqueous pore model fit of *k*_cat_ ratio data and estimated pore sizes for PC and EX; inset: sigmoidal fit and estimated pore sizes of 4.4 and 2.7 nm for PC and EX, respectively. Horizontal errors bars (*n* = 3) are the standard deviation values for the hydrodynamic radii. Vertical errors bars (*n* = 3) reflect the standard deviation for the turnover values (*k*_cat_).

From structural models, the three morphologies of P22 VLPs all contain distinctly different pore sizes (shown in Fig. 3a–c modeled using UCSF Chimera^48^). The PC morphology (PDB: 2xyy) has pores that are modeled to be approximately 4.2 × 1.9 nm, positioned at the center of the hexamers (Fig. 3a). Upon transformation to the expanded (EX) form (PDB: 5uu5), the pores become much smaller due to a conformational shift of the CPs (Fig. 3b). Lastly, the WB structure (PDB: 3iyh) has large, approximately 10 nm, pores (Fig. 3c) as well as smaller pores present at the center of hexamers. Although these pore sizes have been modeled in the cryo-EM reconstructions, such structural models are static averages and reflect neither the potential dynamic nature of these particles (and pores) in solution, nor account for contributions from the flexible side chains.

**Fig. 4:**
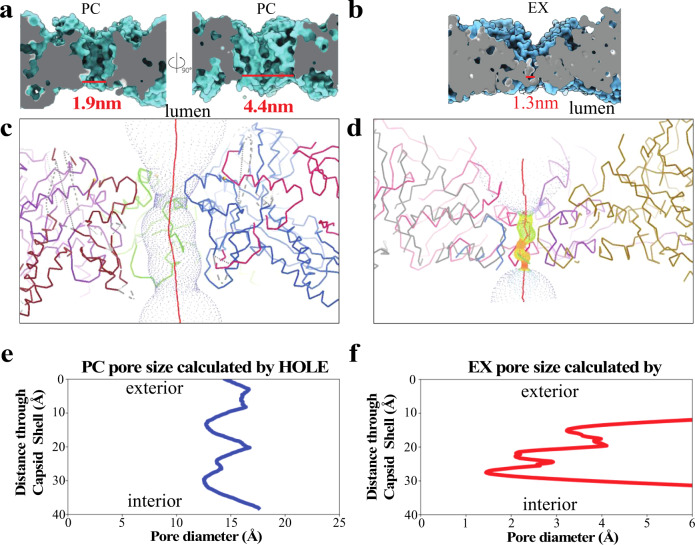
Using HOLE to model P22 capsid pores for procapsid (PC) and expanded (EX) morphologies. **a** Image on the left is a model of the theoretical pore size of PC considering the electron density contribution of the amino acid side chains. Image on the left is the theoretical pore size of PC excluding the side chain electron density. **b** A model of the theoretical pore size of the EX capsids including side chain electron density. **c**, **d** Simulated path a sphere would travel when traversing across the P22 capsid shell for PC and EX morphologies, respectively. **e**, **f** A representation of distance traveled across the capsid shell as a function of pore diameter.

To account for the electron density and steric contributions from the side chains around the pores, we then used HOLE to model the pores for the PC and EX P22 capsids (Fig. 4a, b)^49^. This method resulted in average pore sizes of 1.9 and 1.3 nm for PC and EX, respectively. HOLE used a Monte Carlo algorithm to simulate the route a sphere would take in order to squeeze through the pore (Fig. 4c, d) and also calculate a theoretical diameter of the pore as a sphere diffused across (Fig. 4e, f). The effective diameter calculated in Fig. 4e for PC ranged from 1.3 to 1.8 nm, while for EX (Fig. 4f) it ranged from 0.15 to 0.6 nm. These simulations all indicate much smaller pores size when the side chains are considered, versus when they are excluded. To confirm this observation was due to the side chains rather than the differences in the software programs, we also modeled the PC pore excluding the side chains (Fig. 4a). Without the side chains, HOLE shows a diameter of 4.4 nm, which is very close to the pore sizes calculated using Chimera. These results further underlined the need to determine the effective pore size a small molecule encounters when diffusing across the P22 particle.

AdhD enzymes within the P22 morphological variants behaved as a catalytically active macromolecular bait. A reaction mixture containing both the modified NADH-Neg and the substrate, acetoin, was used. Thus, NADH molecules able to traverse the capsid shell and access the inside of the VLPs would be oxidized to NAD^+^-Neg and the reaction would proceed as shown in Fig. 3d. If NADH-Neg was not able to access the interior, there would be no reaction. The oxidation of NADH-Neg was monitored by the decrease in the characteristic absorbance of NADH at 340 nm. To account for any differences in the enzyme reaction with different NADH-Neg generations, the activities of the encapsulated enzymes were compared to that of free enzymes with the same substrates and the kinetic parameters are presented as a ratio of free/encapsulated.

Monitoring the activity of the NADH substrates clearly showed that diffusion is severely inhibited when the substrate size becomes too large to traverse the PC and EX capsids (Fig. 3e). The *k*_cat_ values obtained from the PC and EX samples showed a very sharp transition with a significant drop in turnover for reactions with generations >NADH-Neg_3.5_ for PC, and >NADH-Neg_2.5_ for EX (Fig. 3e). These data suggest that after NADH-Neg_3.5_ and NADH-Neg_2.5_ for PC and EX, respectively, the diffusion of substrates across the capsid was inhibited and have approached the size limitations of the pores. The ratio of turnover numbers showed a similar trend for the AdhD-SP_free_ and WB samples that was distinct from PC and EX samples. With increasing size of the dendrimer, the apparent *k*_cat_ values of the free and WB-enzyme decreased following the same downward slope across all dendrimer sizes. With increasing dendrimer generations, the modified NADH molecules became larger and the measured *K*_M_ values followed an increasing trend, although there were no significant differences between AdhD-SP_free_ and AdhD-SP encapsulated in the three different particle morphologies. This observation suggests that as the size of NADH increased, substrate binding to the enzyme active site was perturbed, but encapsulation of the enzyme did not significantly alter the interaction between NADH molecules capable of accessing the active site. The kinetics using AdhD-SP_free_ and the three morphologies of P22 (PC, EX, and WB) are shown in Supplementary Fig. 17, along with the extracted apparent *k*_cat_ and *K*_M_ parameters in Supplementary Fig. 18. The values established using NADH and NADH-Neg_0.5_ for free, PC, EX, WB were comparable to those of AdhD-SP_free_, suggesting free and uninhibited diffusion of these small molecules across the capsid shell.

To visualize the size selectivity observed in these results, Fig. 3e shows the ratio of the apparent *k*_cat_ values obtained using SP-AdhD-SP_free_:P22-AdhD versus the measured size (*R*_*H*_) of the modified NADH-Dendrimers. The PC and EX traces each showed drastic deviations from the AdhD-SP_free_ above a discrete size threshold where the NADH-Neg generations became too large to diffuse through the pores readily. The red trace shows the ratio of ~1 between AdhD-SP_free_ and PC-AdhD for NADH-Neg_0.5–3.5_ but increased significantly for NADH-Neg_4.5–5.5_ indicating that the turnover for the AdhD-SP_free_ enzyme was much higher than the AdhD enzymes encapsulated within the PC. These data suggested that the size limitation for molecules traversing across PC is in the range of 4.2–6.2 nm. The measured threshold for diffusion across the EX particles was smaller than that of PC and WB (between 2 and 4 nm) as shown in the green trace where the turnover rates decrease significantly for generations >NADH-Neg_2.5_. The blue trace shows a constant ratio ~1 between AdhD-SP_free_ and WB-AdhD across all dendrimer generations, indicating that the turnover values remained constant for free and encapsulated enzymes and that diffusion into the capsid was not limited by the pores of WB. This result is consistent with expectations since the largest NADH-Neg_5.5_ conjugate is ~7.5 nm and should therefore easily gain access to the encapsulated enzyme.

The effective pore sizes of the PC and EX P22 particles were determined using two different mathematical models of the kinetic data, both of which resulted in comparable pore sizes for the two morphologies. We did not estimate the pore size of the WB particles because we did not approach its pores size limitation. A permeability function, previously used to model passive diffusion across the nuclear pore complex, was used to determine whether size threshold for permeation across P22 followed behavior more consistent with a rigid or a soft (dynamic) barrier^13^. A soft barrier would be indicated by a linear response between *k*_cat_ ratios with increasing NADH size due to significant pore size fluctuations, whereas a rigid barrier would have a sharp increase in *k*_cat_ (due to rigid diffusion limitations) when approaching the pore size threshold^50,51^. The equation used to model the P22 pore (Methods) took into consideration the theoretical pore size, the radius of the NADH-Neg molecules, the friction between the NADH-Neg molecules as they move across the pore wall, and steric hindrance between the pore and the diffusing NADH-Neg as they approached the pore size and predicted the probability of a NADH-Neg molecule diffusing across that pore. The data shown in Fig. 3b were fit by adjusting different pore sizes (*r*_*0*_) within the equation and the best fit was obtained from a pore radius of 2.2 nm for the PC and 1.35 nm for the EX. It should be noted that the last points from the higher generation dendrimers were removed from the fit. In an ideal system, once the diffusing molecules have reached the pore size limit, the *k*_cat_ values in the P22 nanoreactor experiment would have approached zero, while the ratio of AdhD-SP_free_ *k*_cat_:P22 AdhD *k*_cat_ would become infinitely large. Our data revealed encapsulated *k*_cat_ value approaching zero up to the threshold before reaching a plateau (a slope of zero), which we attribute to the inhibition of NADH accessing the active site. In the model system the slope of the curve approached an undefined value where the *x*-axis coordinates no longer increased. Since our *x*-axis values would have continued to increase as long as the points for larger dendrimer generations were plotted, the points corresponding to slope = 0 were removed for this fitting. To verify that the prediction from this fitting was correct, Fig. 3e and the inset in Fig. 3f show a sigmoidal fit, with estimated pore threshold sizes of 4.4 and 2.8 nm for PC and EX, respectively^52^. We have also modeled the trend that would be followed if P22 particles contained a soft barrier, where the pore sizes undergo large fluctuations (Supplementary Fig. 27). This model resulted in *r*^2^ values of 0.59, indicating a poor fit and therefore unlikely that P22 pores represent a soft barrier. On the other hand, the rigid pore model provided a reasonable fit for our data suggesting that the pores of EX and PC P22 VLP are surprisingly rigid.

### Probing the effects of charge on diffusion of molecules across the P22 VLP

In order to determine the effects of electrostatics on the gating of molecules passing across the VLP barrier, we synthetically neutralized the negative charges on the dendrimers and also varied the ionic strength of the buffer. For this study positively charged dendrimers were not used because at the concentrations of the NADH-Neg conjugates required for these experiments, the particles are known to coalesce into a bulk aggregated material^53,54^.

**Fig. 5:**
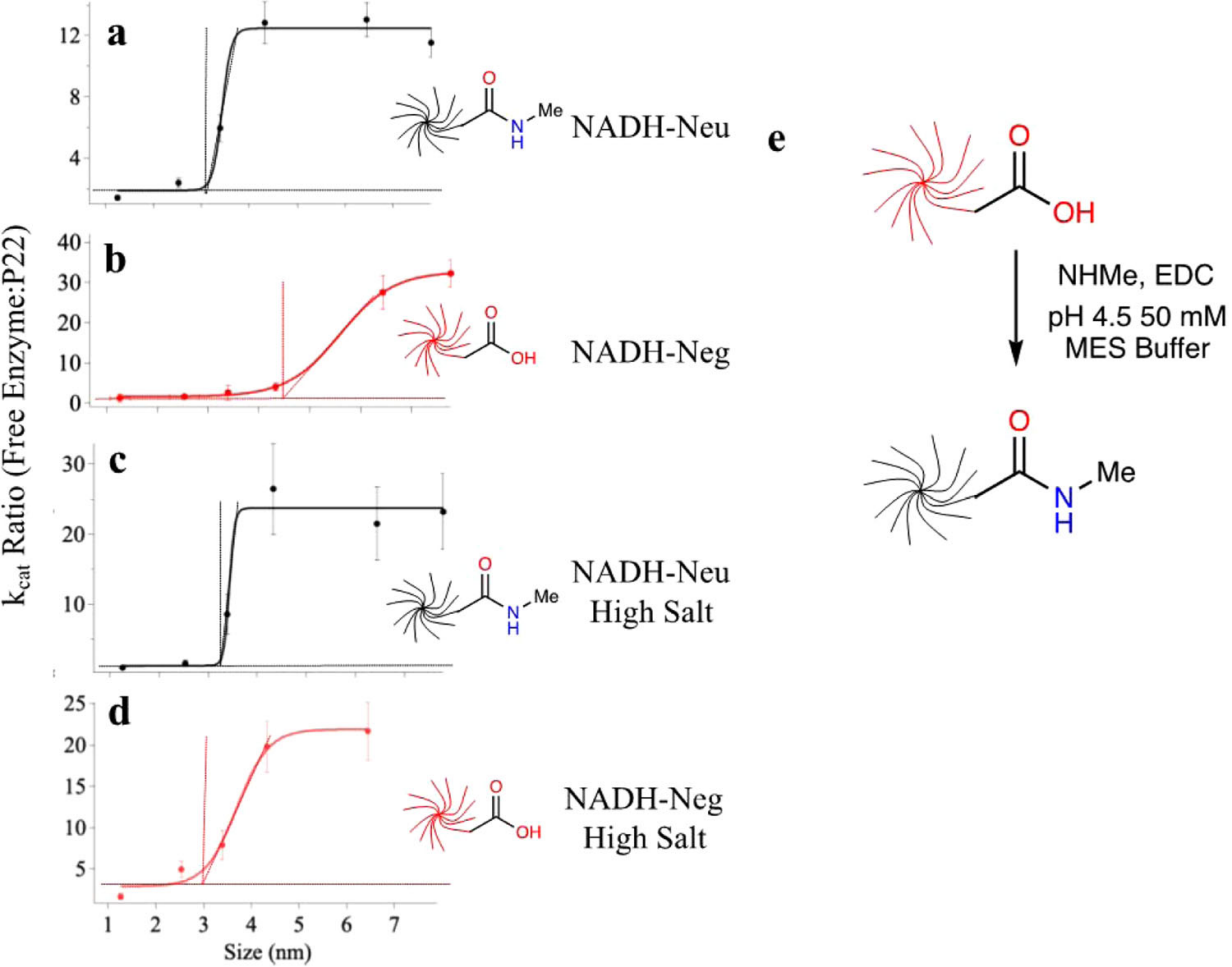
Effects of electrostatics on the effective pore size of P22 PC VLPs. All graphs (**a**–**d**) are portrayed as the ratio of extracted *k*_cat_ values for free AhdD-SP enzymes and those encapsulated inside P22 particles versus diameter of NADH conjugates with sigmoidal fits. The data acquired using NADH-Neu are depicted in black, while with NADH-Neg in red. **a** Effective pore size of 3 nm using neutral dendrimers in pH 7, 50 mM sodium phosphate, 100 mM sodium chloride. **b** Effective pore size of 4.3 nm using negative dendrimers in pH 7, 50 mM sodium phosphate, 100 mM sodium chloride. **c** Effective pore size of 3.2 nm using neutral dendrimers in pH 7, 50 mM sodium phosphate, 400 mM sodium chloride. **d** Effective pore size of 3.0 nm using negative dendrimers in pH 7, 50 mM sodium phosphate, 400 mM sodium chloride. The limits of the *y-*axis in **a**–**d** are not uniform in order to more accurately compare the activity differences between the P22-AdhD and the free enzyme with respect to the size of the NADH conjugates. **e** Scheme of reaction used in order to neutralize the negative charges on the dendrimers. Error bars = standard deviation (*n* = 3).

Charge neutralization of the NADH-Neg molecules was achieved using the reaction outlined in Fig. 5e, where the carboxylate-terminated dendrimers were activated with EDC and methyl amine was added to form terminal amides forming NADH-Neu. DLS and zeta potential were measured in order to confirm the size-integrity of the molecules and verify that the negative charge had been altered. DLS revealed that size of the dendrimers remained comparable after the reaction, and the dendrimer population remained monodisperse after purification (Supplementary Fig. 19). Measurements of zeta potential showed that the new dendrimers were no longer negatively charged and became slightly positively charged (Supplementary Fig. 19) across all generations, likely due to the protonation of some of the internal tertiary amines^55^.

The kinetic parameters (Michaelis–Menten constants) and the ratios of SP-AdhD_free_ and P22-AdhD were determined and plotted for the NADH-Neu dendrimer, the results of which surprisingly indicated a smaller effective pore size, compared to the effective pore size determined using NADH-Neg. The *k*_cat_ ratios were once again used to assess the differences in diffusion across the P22 barrier (Fig. 5a, b). The kinetic ratio significantly increased around NADH-Neu_3.5_ indicating that the turnover number using the PC-AdhD decreased, which we attributed to the inability of the NADH to diffuse across PC P22. Using the same sigmoidal fitting model as was previously used to compare P22 PC, EX, and WB, the theoretical pore size diameter decreased from 4.4 to 3 nm when using the NADH-Neu molecules. Data from the kinetic assays, *K*_M(Acetoin)_, *k*_cat(Acetoin)_, and kinetic efficiency using AdhD-SP_free_ and PC-AdhD with the neutral NADH-Neu can be found in Supplementary Figs. 20 and 21. The apparent *K*_M_ values using both free and encapsulated enzymes were higher when using the NADH-Neu molecules than they were when using NADH-Neg and *K*_M_ did not increase with increasing dendrimer generations. This suggested that the neutral species might have blocked acetoin docking at the enzyme’s active site even at small dendrimer generations, in contrast to the size of the dendrimer that had a smaller effect. The lack of steric effects can likely be attributed to the flexible linker between NADH and the dendrimers.

To further explore the contribution of charge on the effective pore size, kinetic experiments were completed using NADH-Neg and NADH-Neu in high salt (HS) buffer (Supplementary Figs. 22 and 24) to shield electrostatic interactions. Analysis of the *k*_cat_ values for all four conditions (NADH-Neg, NADH-Neu, NADH-Neg_HS_, NADH-Neu_HS_) yielded unexpected results in that NADH-Neg exhibited the largest effective pore size. The turnover number ratios obtained with free and encapsulated AdhD-SP are displayed in Fig. 5 with sigmoidal fits to compare the effective pore sizes. Under HS conditions the effective pore sizes were estimated to be 3.3, 3.0, and 3.0 nm using NADH-Neu_HS_, NADH-Neu, NADH-Neg_HS_, respectively. However, the pore size observed using NADH-Neg was 4.3 nm, suggesting that the electrostatic potential of the P22 VLPs and their interactions with the diffusants also play a role in guiding molecules across its barrier, especially as the size of the molecule approached the limitations of the pore. This was especially evident with Generation 3.5 molecules that were able to access the interior of P22 when in the form of NADH-Neg_3.5_, but not the other three conditions. Previous studies have also demonstrated that electrostatic effects play a role in diffusion across a porous protein cage^56,57^; however, in our case, the molecules that diffuse across more readily carry the same net charge as the protein cage.

The apparent *K*_M,HS_ using free and encapsulated enzymes behaved similarly when using both NADH-Neg and NADH-Neu, with a slight net increase in *K*_M,HS_ with increasing dendrimer size, which is a similar trend to what we saw with NADH-Neg in lower ionic strength buffer. The apparent *K*_M,HS,(Acetoin)_, *k*_cat,HS,(Acetoin)_, and efficiency can be found in Supplementary Figs. 23 and 25. In some enzymes the electrostatic surface potential can play a significant role in guiding substrates towards the active site and has been well studied^58–61^. The electrostatic surface potential of a previously published homology model of the AdhD enzyme (Supplementary Fig. 26) was computed and the enzyme was found to overall carry a net negative surface charge with positively charged patches around the entrance to the active site. The isoelectric points were also computed to be 6.59 and 5.93 for AdhD-SP and AdhD, respectively. Thus, the surface potential map and the pI values were in good agreement. When using the NADH-Neg molecules (Supplementary Fig. 18) a lower *K*_M(Acetoin)_ was observed compared to values observed with NADH-Neu (Supplementary Fig. 21), which might be attributed to the NADH-Neg blocking acetoin’s access to the active site less than the slightly positively charged NADH-Neu molecules, due the repulsive electrostatic interactions. This is further supported by the apparent *K*_M_ values of acetoin collected using NADH-Neg_HS_, NADH-Neu, and NADH-Neu_(HS)_ all of which have higher observed *K*_M_ values compared to those collected using NADH-Neg. The increase in *K*_M(Acetoin)_ between NADH-Neg_0.5–5.5_ was lower for the reactions in which charges were either screened or neutralized (NADH-Neg_HS_, NADH-Neu, NADH-Neu_(HS)_). This observed phenomenon was likely due to a decrease in electrostatic contributions from the positively charged patches on the AdhD to the NADH-Neg.

**Fig. 6:**
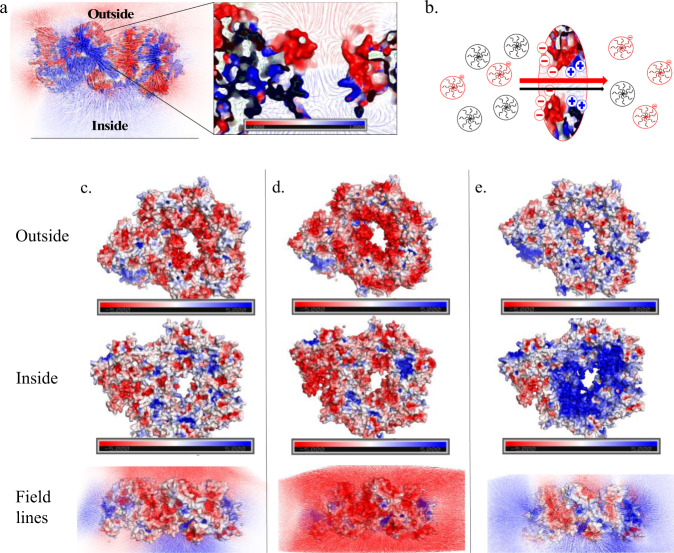
Electrostatic maps of P22 pores. **a** Electrostatic surface potential of the wtP22 PC asymmetric unit with electric field lines with negative and positive surface potential in red and blue, respectively. Maximum and minimum units are 1 to −1 kT/e. **b** Cartoon of dendrimers diffusing across the P22 pore. **c**, **d** Comparison of P22 CP electrostatic surface outside (top), inside (middle), and field lines (bottom) for **c** wt, **d** K184Q/R203S, and **e** D143N/D357N.

Upon modeling the electrostatic potential of P22 particles, we saw that there were positively charged regions around the interior of the P22 pores that could facilitate interactions between NADH-Neg and the pores, even though the net charge of the P22 particle is negative. The results showing the charge density around the pores of the PC P22 can be found in Supplementary Movie 1 and Fig. 6a, c. Although side chain information was uniformly truncated in the PC structure deposited in the PDB, at 3.8 Å resolution there is high confidence in the protein backbone location and rudimentary side chain positioning. Consequently, we used Coot software to manually add probable rotamer conformers in order to generate an electrostatic map of the molecular surface of P22 using PyMOL and APBS. Although the exact rotamer positioning is uncertain, the overall surface charges likely remain valid using this technique. The asymmetric unit with this encoded information is shown in Fig. 6a, c and in Supplementary Movie 1. With this electrostatic information, the charge density around the outer surface of the pore appeared negative, while the charge density on the inside surface of the pore appears positive (blue). The inset in Fig. 6a shows the charge distribution across the pore, along with the electric field lines.

The electric field lines provide the likely path a charged molecule might follow within the electrostatic field, a phenomenon previously observed in ferritin protein cages and acetylcholine esterase^58,61–63^. The insert in Fig. 6a provides a model for the electric field surrounding the pore area, where the very negatively charged exterior transitions into a positively charged interior. This generates a charge gradient between the exterior and interior of the capsid. At the ionic strength conditions used in these reactions, it is likely that the dendrimers can get close to the capsid before experiencing the electric field. Once within the field, we hypothesize that the charged molecules will diffuse away from the point charges on the VLP following the trajectory of the field lines, until encountering the positively charged field on the interior region of the pore, which would effectively pull the molecules into the particle. With an increase in ionic strength, the Debye length decreases, effectively weaking the electrostatic interactions between the pores and the NADH molecules. This was supported by the data that showed the NADH-Neg_HS_ and NADH-Neu_HS_ molecules having very similar trends in activity and the same calculated effective pore sizes.

To test the validity of our hypothesis, we made two different mutants of the P22 CP subunits that, when assembled into the P22 VLP capsid, changed the charge distribution on either the particle exterior or interior surfaces. Each new mutant contained two mutations: D143N/D357N and K184Q/R203S. The first set of mutations removed negative charges on the exterior both around the pore and regions further away, while the latter mutation removed two positive charges on the interior of the capsid at the region close to and within the pore. The particles were characterized using the same methods as the particles described in earlier sections of Results and discussion (Methods and Supplementary Figs. 28–30). Zeta potential measurements revealed that the D143N/D357N mutant had a less negative zeta potential (−19.8 mV) compared to wt P22 (−23.8 mV), while the K184Q/R203S mutant exhibited a more negative zeta potential (−25.2 mV). The electrostatic surfaces and their respective field lines were also calculated, showing a very clear difference in charge distribution both on the interior and exterior surfaces (Fig. 6d, e). D143N/D357N remained positively charged on the inside; however, the exterior showed a much less negative surface (Fig. 6e) compared to wt P22 (Fig. 6c). The field lines for K184Q/R203S reflected a much more significant change to the landscape where the positively charged patches seen on the inside in wtP22 completely disappeared (Fig. 6d).

**Fig. 7:**
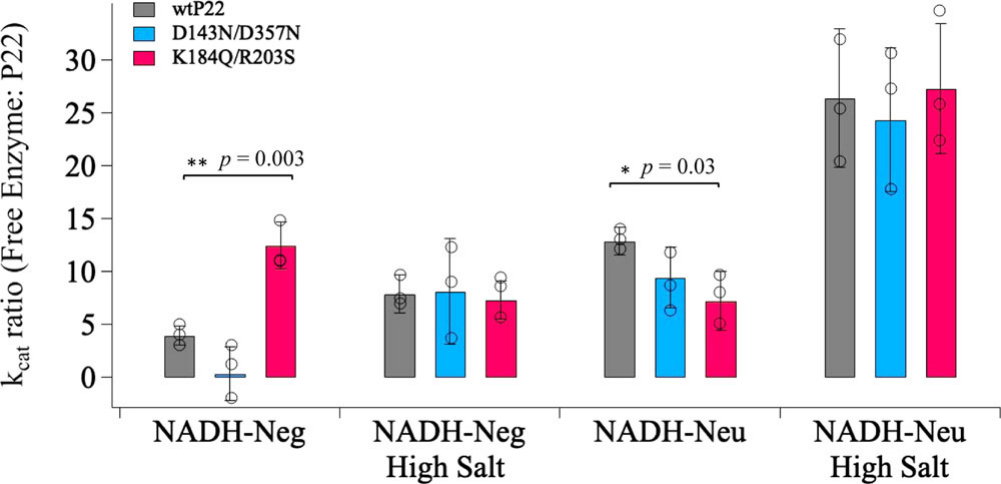
Bar graph comparing *k*_cat_ ratios of enzymes encapsulated within wtP22 (gray), D143N/D357N (blue), and K184Q/R203S (red) with NADH-Neg_3.5_, NADH-Neg_3.5(HS)_, NADH-Neu_3.5_, and NADH-Neu_3.5(HS)_. The most statistically significant differences among the P22 variants are seen with NADH-Neg conjugates with a *p* value of 0.003 when comparing activity ratios of wtP22 to K184S/R203S using two-tailed *t* test, where calculated *t* values far exceeded expected values at the 95% confidence level. *P < 0.05, **P < 0.005, and not significant when *P* > 0.05. *n* = 3, 4 d.f. Error bars = standard deviation.

Testing the diffusion limits of these mutants revealed that when the charges of the positive inner pore were neutralized, molecules previously capable of diffusing across the pore were no longer able to do so. For this experiment, we chose generation NADH-Neg_3.5_ and NADH-Neu_3.5_ because in earlier experiments we saw that the negative substrate could access the interior of the wtP22, while the neutral substrate could not. The kinetic plots and extracted kinetic constants using NADH and each of the mutants can be found in Supplementary Fig. 31, while the data collected using NADH-Neg_3.5_ and NADH-Neu_3.5_ are shown in Fig. 7. The NADH-Neg_3.5_ showed very little activity when used together with the negative mutant (K184Q/R203S), while with the positive mutant (D143N/D367N) showed slightly enhanced activity, albeit with some inhibition compared to wtP22 (Supplementary Fig. 31a and Fig. 7). These data are consistent with our earlier hypothesis in that the positive charges located within the pore facilitate the diffusion of charged molecules across the pore and their removal in the K184Q/R203S mutant inhibited the uptake and oxidation of the NADH-Neg_3.5_. The activity ratio (*k*_cat_ for free:*k*_cat_ encapsulated) for K184Q/R203S with NADH-Neg_3.5_ is much higher compared to values collected on wtP22 (Fig. 7). In contrast the ratio values for D143N/D357N are slightly lower than for the wtP22 indicating more access of the substrate across the pore in response to the diminished negative charge of the capsid exterior. These charge-based differences disappeared when the ionic strength of the medium was increased, due to charge screening, where both mutants and wtP22 capsids exhibited roughly the same activity ratios (Fig. 7).

In summary, this study allowed us to systematically probe molecular diffusion across the porous barrier. Using P22 VLP capsids as a model system we determined the pore sizes for two different particle morphologies; the EX particles (2.7 nm) and PC (4.4 nm). Another morphology, the WB, allowed molecules up to 7.5 nm to diffuse freely across the capsid boundary and served as a control where the barrier to small molecule diffusion was removed. Although there has been speculation on the dynamic nature of these VLPs and simulations that have predicted that the pores may undergo large fluctuations^40,41^, the lack of high-resolution structural data had limited our knowledge. Our data showed that the molecular cut-off size was close to the size of the pores calculated using the available cryo-EM model. However, when considering the side chains of the residues lining the pores, the data suggested that the pores of P22 are dynamic where molecules twice the theoretical size were able to diffuse across. The effective pore size is highly dependent on the charge of the diffusing molecule and can be tuned by neutralizing the molecular charge or increasing the ionic strength of the reaction buffer; both of which resulted in a decreased effective pore size. These are interesting findings considering that the exterior of P22 is known to be negative and it was the negatively charged dendrimers that were able to diffuse across more readily. Upon analyzing the electrostatic potential around the pores, we suggest a charge-driven mechanism for the negatively charged molecules to diffuse across, where the charge gradients around the pores guide the molecules diffusing across. Porosity is a crucial structural feature present in many biological compartments and often an important principle when designing new functional materials as a way to segregate components. Here we showed how the pores can drastically affect enzymatic activity, perhaps providing further insight into how their presence contributes to the specificity of catalysts in biological systems and those that are synthetically derived. We have presented a systematic study of the molecular porosity and permeability of a compartment and determined the diffusion limitations across a model capsid boundary in terms of both size and charge.

## Methods

### Site-directed mutagenesis protocol for D143N/D357N and K184Q/R203S P22 CP

The sense and anti-sense primers (Supplementary Table 1) were designed to incorporate the desired mutations and are displayed in Supplementary Information. The two mutations were inserted into each construct simultaneously by mixing four primers in each reaction setup. The setup for each reaction was as follows: 6.6 μL of PCR grade water, 0.6 μL of each primer (10 μM) to a final concentration of 0.3 μM, 1 μL of 10 ng pRSF-Duet plasmid containing the CP gene, and 10 μL of KOD Hot Start Master Mix (0.04 U/μL). The PCR cycling followed the recommended conditions for the KOD polymerase: (1) polymerase activation (2 min at 95 °C), (2) denaturation (2 min at 95 °C), (3) annealing at the lowest Tm °C for 10 s, (4) extension for 125 s at 70 °C (25 s/kbp); followed by steps 2–4 cycled 40 times. Before transforming into cells, the PCR products were incubated with dNTPs for 4 h at 37 °C.

### Protein expression and purification: wtP22 AdhD-SP, D143N/D357N P22 AdhD-SP, K184Q/R203S P22 AdhD-SP, and free AdhD-SP

Two vectors, with different antibiotic selection, were simultaneously transformed into BL21 Electrocompetent Cells. A pRSF-Duet vector contained the CP gene, while pBAD contained the AdhD-SP cargo. The genes were expressed on LB medium at 37 °C with kanamycin and ampicillin to select for pRSF-Duet and pBAD, respectively. L-arabinose (13.3 mM) was used for induction of AdhD-SP once the cells reached OD_600_ 0.5–0.7. After a 4-h incubation at 37 °C, isopropyl β-D-thiogalactopyranoside (0.5 mM) was used for induction of CP. Following the second induction, the culture was allowed to continue growing overnight at room temperature. In the instance where free AdhD-SP was expressed (without CP), the cells were harvested after the first incubation period following arabinose induction, while for P22 AdhD after the overnight incubation. The recovered cell pellets were resuspended in 50 mM sodium phosphate, 100 mM sodium chloride, pH 7.0. In order the lyse the cells, the samples were frozen and thawed, incubated with an enzyme cocktail containing lysozyme, DNase, and RNase at room temperature for 30 min, followed by sonication for 2 min. The suspension was centrifuged at 12,000 × g for 45 min at 4 °C using Sorvall Legend XTR Centrifuge with the F15-8x50cy FIBERLite rotor. The supernatant was recovered and ultra-centrifuged through a 35% (w/v) sucrose cusion at 215,619 × g for 50 min. This step resulted in a pellet containing the P22 particles, which were then resuspended in 50 mM sodium phosphate, 100 mM sodium chloride, pH 7.0 buffer, and purified over a S-500 Sephadex (particle size 25–75 μM; GE Healthcare Life Sciences) column using BioRad Biologic Duoflow FPLC. The relevant fractions were then pooled and concentrated for further use. Free AdhD-SP contained a poly-histidine tag and therefore was isolated using Roche Ni-NTA affinity column where an imidazole gradient was introduced to the mobile phase to elute the protein. The concentrations of P22 AdhD and AdhD-SP were determined using absorbance at 280 nm with molar extinction coefficients of 44,920 M^−1^ cm^−1^ (CP) and 61,310 M^−1^ cm^−1^ (AdhD-SP).

### Size-exclusion chromatography with multi-angle light scattering and refractive index detection

MALS coupled with SEC was used to determine the molecular weight of all the constructs. The samples (25 μL/each) were introduced to the mobile phase of an Agilent 1200 HPLC using an autoinjector and separated over WTC-200S 5 μM, 2000 Å, 7.8 × 300 mm column. A 16-angle MALS detector (HELEOS-Wyatt Technology Corporation) and Optilab rEX refractometer (Wyatt Technology Corporation) then detected the scattering and concentration of the injected samples. Astra 5.3.14 software (Wyatt Technology Corporation) was used for data analysis with the following equation used to compute the molecular weight:

1 $$M=\frac{R\left(\theta \right)}{\frac{{4\pi }^{2}{n}_{0}^{2}}{{N}_{A}{\lambda }_{0}^{4}}{\left(\frac{{dn}}{{dc}}\right)}^{2}{cP}(\theta )}$$

Where $R\left(\theta \right)$ is the excess Rayleigh ratio from solute, ${n}_{0}$ is the solvent refractive index, ${N}_{A}$ is Avogadro’s number, ${\lambda }_{0}$ is the wavelength of incident light, $\left(\frac{{dn}}{{dc}}\right)$ is a material-specific refractive index increment where in the case of proteins the value is 0.1850 mL/g, $M$ is the molar mass, $c$ is the solute concentration in w/v, and finally $P(\theta )$ is the form factor.

### Transmission electron microscopy

For obtained microscopy images of the particles, the samples were prepared with a 0.1 mg/mL concentration and 10 μL was applied to formvar coated grids (Electron Microscopy Sciences). The droplet was covered and allowed to incubate at room temperature for 5 min. The droplet was removed using filter paper and grid was washed with 10 μL of distilled water. The grids were then incubated with 10 μL of 1% uranyl acetate for staining, and subsequently removed after a 5 min incubation. JEOL 1010 transmission electron microscope was used for imaging with a 100 kV accelerating voltage.

### Agarose gel shift assay

The native gel for the shift assay was prepared by adding 0.4 g of agarose to 50 mL of 40 mM Tris, 5 mM acetate, 1 mM EDTA, pH 8.2 to result in a 0.8 % (w/v) concentration. Bromophenol blue-containing loading buffer was added to all samples prior to loading. The shift assay was run for 2 h at 75 V with the running buffer matching the gel buffer above. The gel was stained by Coomasie blue, rinsed with water, and incubated with destaining solution. UVP MultDoc-IT Digital Imaging System was used to obtain the gel images.

### P22 expansion to wiffle-ball morphology

The P22 protein concentration was adjusted to 1 mg/mL using 50 mM sodium phosphate, 100 mM sodium chloride, pH 7.0 buffer, and heated at 75 °C for 20 min. The samples were cooled and usually a precipitate, containing aggregated proteins, formed that was removed using centrifugation. Ultracentrifugation (215,619xg-Sorvall WX Ultra Series) was used to recover the intact WB particles.

### Expansion using SDS

The P22 protein concentration was adjusted to 1 mg/mL using 50 mM sodium phosphate, 100 mM sodium chloride, pH 7.0 buffer, and a 7 mM concentration of SDS solution was prepared in the same buffer. The SDS solution was added slowly to the P22-containing solution to result in final concentrations of 0.5 mg/mL of protein and 3.5 mM SDS. Incubation proceeded for 5 min at room temperature and then immediately ultra-centrifuged twice to remove SDS and any broken particles.

### CsCl gradient purification

To ensure maximum homogeneity of P22 particles, all samples were also purified over a CsCl gradient. The gradient was prepared using 1.2 and 1.4 g/mL CsCl solutions in sodium phosphate buffer and then mixed using Biocomp Gradient Master 108 preset setting for 12–14% (w/w). Protein concentration was adjusted to be between 8 and 10 mg/mL and 750 μL of solution was carefully added to the top of the mixed gradient. The samples were then ultra-centrifuged 287,472 × g for 2 h and resulted in a band midway through the sample tube containing the particles.

### Monitoring activity of wtP22 AdhD-SP, D143N/D357N P22 AdhD-SP, K184Q/R203S P22 AdhD-SP, and free AdhD-SP

Activity assays were carried out on Agilent 8454 Uv-Vis Spectrophotometer at room temperature. The buffer used for kinetics studies was 50 mM sodium phosphate, 100 mM NaCl, pH 7.0, unless otherwise specified. For the AdhD activity assay, P22 PC, EX, and WB, encapsulating SP-AdhD cargo, were adjusted to a concentration of 650 nM of encapsulated SP-AdhD, and the substrate (Acetoin) concentration was varied from 1 to 200 mM. The concentration of NADH variants was 220 μM (*ε*_340_ = 6.22 mM^−1^cm^−1^). It is likely that this concentration is not accurate due to the NADH modifications; however, since we were mainly focused on comparing free to encapsulated, this was not significant. There was a total of seven different concentrations for each kinetic assay, and each reaction shown in the Supplementary Information documents was repeated in triplicate. To ensure that minimal free enzyme was present in the samples before monitoring kinetics of the encapsulated enzymes, the P22 solution was ultra-centrifuged and purified over a Ni-NTA column to remove any his-tag equipped free AdhD-SP enzymes before each batch of reactions and used immediately. Kinetics assay done at higher ionic strength (HS) was done in pH 7 50 mM sodium phosphate, 400 mM sodium chloride buffer. Everything else was kept constant. The Michaelis–Menten constant (*K*_M_) and the turnover rate (*k*_cat_) values were extracted using a nonlinear regression Eq. (2).

2 $$f(C)=\frac{v[C]}{{K}_{\rm{M}}+[C]}$$

where *[C]* refers to the substrate concentration, since the concentration of NADH remained constant, this is referring to the concentration of acetoin. The *v* variable refers to the maximum velocity at a given *[C]*. The Michaelis–Menten constant, *K*_M_, is the *[C]* at 1/2 *v*_max_.

### Aqueous pore model prediction

The model used here was adapted from Renkin et al. and Timney et al.^13,50^. In these equations, the pores in the particles were assumed to be cylindrical and the dendrimers traversing across were considered as spheres. There were two equations combined that considered two different scenarios. Equation (3) describes a situation in which the dendrimers pass across the VLP pores without interacting with the edges of the pore.

3 $$A={{A}_{0}\left(1-\frac{r}{{r}_{0}}\right)}^{2}$$

The effective pore area (*A*) can be calculated by multiplying the theoretical cross section of the pore (*A*_*0*_) by the dendrimers probability to enter the pore: ($1-\frac{r}{{r}_{0}}$)^2^, where *r* is the radius of the dendrimer and *r*_*0*_ is the radius of the pore. The second scenario corrects for the friction that may arise between the pore of the particle and the dendrimers:

4 $$\frac{A}{{A}_{0}}={1-2.104\left(\frac{r}{{r}_{0}}\right)+2.09\left(\frac{r}{{r}_{0}}\right)}^{3}{+.95\left(\frac{r}{{r}_{0}}\right)}^{5}$$

Multiplying the steric effects of the dendrimers entering the pore and the frictional contribution of the interaction between the VLP pore wall and the dendrimer (4), we can calculate the permeability coefficient (*P*):

5 $$P=\left({\left(1-{\frac{r}{{r}_{0}}}\right)}^{2}\right)\left(\left(\right.1-2.104\left({\frac{r}{{r}_{0}}}\right)+2.09{\left({\frac{r}{{r}_{0}}}\right)}^{3}+.95{\left({\frac{r}{{r}_{0}}}\right)}^{5}\right)$$

The permeability coefficient describes the rate of diffusion (cm/s), while we measured the rate of enzyme turnover as a function of dendrimer size. Because we directly compared free and encapsulated enzyme, the ratio between the two directly correlated with the diffusion across the porous membrane and corrected for effects arising from the dendrimer-NADH conjugates accessing the active site. Rearranging (5), we fit the data corresponding to the *k*_cat_ ratio of free: encapsulated as a function of dendrimer size using (6).

6 $$f(r)=\frac{P}{\left({\left(1-\frac{r}{{r}_{0}}\right)}^{2}\right)\left(\left(\right.1-2.104\left(\frac{r}{{r}_{0}}\right)+2.09{\left(\frac{r}{{r}_{0}}\right)}^{3}+.95{\left(\frac{r}{{r}_{0}}\right)}^{5}\right)}$$

### Sigmoidal curve fitting for pore size prediction

The sigmoidal curve is typically used as a dose-response curve to identify cooperativity within a system and predict the likelihood of a binding event. In our system it was used to predict the likelihood of a substrate accessing the encapsulated enzyme. The basal region of the curve showed the expected linear response where increasing substrate size slowly decreases the *k*_cat_ when measured using the encapsulated enzyme. This was likely a result of friction between the pore wall and the substrate. The asymptotic, or the maximum, region of the curve provided us with the sizes of substrates no longer gaining access to the enzyme. The slope of the fast growth phase, or the Hill Slope, and slow growth phase base allowed us to calculate the inflection point and predict the pore size. We interpret the transition between the slow growth and fast growth phases (inflection point) as a shift from substrate diffusion across the pore being limited by friction to a system limited by sterics when the substrate size approached the pore size. Equation (7) was used to fit the data and calculate the required values,

7 $$f\left(d\right)={\rm{Base}}+\left(\frac{{\rm{Max}}}{1+{{\rm{e}}}^{\left(\frac{{d}_{{\rm{half}}}-d}{{\rm{Slope}}}\right)}}\right)$$

where *d* was the size of the dendrimer, the base was the value at which the curve crosses the *y*-intercept, max established the maximum value at which the slope approached zero, *d*_half_ is the diameter at the midpoint between the base and max and is used to establish the Hill Slope, labeled as slope in (7). The *d* at which the base line and slope line intersect is called the inflection point, and also the theoretical pore size.

### Electrostatic surface of the pores

To map the electrostatic surface of the PC P22 pores, the coordinates of PDB 2xyy were loaded into COOT where amino acids containing truncated side chains were mutated to the same amino acids with intact side chains. The lowest energy conformer was computed and a modified 2xyy PDB file was established. The structural information from the PDB file was then used to generate a PQR file using APBS online software. This software used the Poisson–Boltzmann calculations to compute an electrostatic map of CP surface. PyMOL was then used to visualize the molecular surface using the PDB generated by COOT and the APBS PQR file. The movie was made using ImageJ software.

### Synthesis of NADH-Neg_xx_ conjugates

#### 8-NAD^+^-Br

The first step of the reaction was the bromination of NAD^+^ at the C8 position of adenine. NAD^+^ (5.00 g, 7.55 mmol) was dissolved in pH 4.5, 50 mM sodium acetate buffer (60 mL) at room temperature. Reaction was stirred under argon as bromine (2.0 mL, 39.05 mmol) was added dropwise to solution. Reaction was allowed to proceed overnight. Note: the reaction still worked when completed without argon. We have found that a precipitate formed upon the addition of bromine that contributed to a lower yield of our product. The completion of the reaction was monitored by changes in the UV absorbance where the λ_max_ shifted from 259 to 265 nm (Supplementary Fig. 3) and further verified using H^1^ NMR through the disappearance of the C8 proton peak (Supplementary Fig. 4)^46^. For this step it was important to protect NAD from electrophilic attack at the C5 position of the nicotinamide; therefore, the oxidized form (NAD^+^) was used. The mixture was washed with carbon tetrachloride until the organic layer showed little to no color. The aqueous phase was recovered and dialyzed into water overnight using a 500 MW cut-off cellulose dialysis tubing (Spectrum Labs). The resulting solution was lyophilized for 48 h and product was recovered as a light-yellow powder (3.09 g, 52% yield). ^1^H-NMR (400 MHz, D_2_O) ${\rm{\delta }}$ 9.25 (s, 1H), 9.10 (d, 1H), 8.75(d, 1H), 8.20 (t, 1H), 8.1 (s, 1H), 6.10 (d, 1H), 5.95 (d, 1H), 4.0–4.5 (m, 9H).

#### 8-NADH-Br

At this point in the synthesis NAD^+^ was reduced to NADH, which is more thermally stable as the C2 and C6 of nicotinamide of NAD^+^ are especially prone to nucleophilic attack, which was important to avoid in the following step. 8-NAD^+^-Br (351.3 mg, 0.487 mmol) was dissolved in 18 mL of 1.3% (v/w) sodium bicarbonate buffer using a two-neck RBF. Solution was stirred while bubbling argon gas through solution for 30 min. Using other neck of RBF, sodium dithionite (175.6 mg) was added while continuing to bubble argon. Resulting mixture was stirred under argon gas for 3 h at room temperature, monitored by the appearance of a peak at 340 nm in the UV spectrum (Supplementary Fig. 3) and verified using H^1^ NMR through the upfield shift of the nicotinamide protons (Supplementary Fig. 5). The remaining sodium dithionite was oxidized by removing the flow of argon and stirring while exposed to air for 1 h. The completion of reaction was monitored by measuring the A_265_ to A_340_ ratio and reaction stopped when the ratio approached ~3. The product was recovered by precipitating with 10× cold acetone (stored in −20 °C) and centrifuging at 4500 × g for 5 min. The supernatant was decanted through a filter. Remaining pellet was dissolved in water and lyophilized for 48 h. Product was recovered as a beige solid (430 mg). ^1^H-NMR (400 MHz, D_2_O) ${\rm{\delta }}$ 8.1 (s, 1H), 6.82 (s, 1H), 6.18 (d, 1H), 5.80 (d, 1H), 4.0–5.0(m, 11H), 2.9 (m, 2H).

#### 8-NADH-NH_2_

8-NADH-Br (321 mg, 0.408 mmol) was added to 6 mL DMSO and heated to 60 °C. In a different flask, 2,2′-(ethylenedioxy)bis(ethylamine) = linker (1.285 g, 8.32 mmol) was dissolved in 6 mL of DMSO and also heated to 60 °C. The solution containing 8-NADH-Br was added to solution containing linker. Resulting mixture was stirred under a condenser at 80 °C for 6 h. Reaction was cooled to room temperature, and the acetone precipitation was repeated by adding 120 mL of cold acetone and precipitate was recovered using centrifugation (4500 × g, 5 min). Pellet was dissolved in water and lyophilized to dry. Powder was recovered as a beige solid (318 mg, 60% yield). Yield was calculated based on A_340_. ^1^H-NMR (400 MHz, D_2_O) ${\rm{\delta }}$ 8.2 (s, 1H), 6.75 (s, 1H), 5.85–6.10 (m, 2H), 5.25 (dd, 1H), 3.8–4.5 (m, 9H), 3.1–3.5 (m, 13H), 2.80 (d, 2H) 1.01 (t, 1H). Calculated HRMS for C_27_H_42_O_16_N_9_P_2_ 810.2225, observed 810.2223. Calculated HRMS for C27H41O16N9NaP_2_ 832.2044, observed 810.2043. (Supplementary Figs. 6 and 7).

#### NADH-Neg_xx_

All synthesis for the conjugation proceeded using the same reaction scheme with varying stoichiometric amounts of dendrimer. The theoretical number of NADH molecules per dendrimer was adjusted using the molar ratios displayed in Table 1. The concentrations of NADH were determined using A_340_. 8-NADH-NH_2_ amount was adjusted to 50 mg (61.6 µmols) and kept constant for all reactions. As a representative example: Gen. 4.5 dendrimer (202 mg, 7.7 µmols, 650 µL) was dissolved in 3 mL of pH 4.7, 100 mM MES buffer. EDC (0.0145 g, 75 µmols) was added to the reaction and stirred for 1 h. 8-NADH-NH_2_ was dissolved in the MES buffer (3 mL) and added to solution containing dendrimers. Mixture was stirred overnight and purified using cellulose dialysis tubing. Molecular weight membrane cutoffs varied from 1500 to 14,000 Da depending on the size of dendrimers. Product was lyophilized and stored at 4 °C until use.

#### NADH-Neu_xx_

All synthesis for the conjugation proceeded using the same reaction scheme with varying stoichiometric amounts of methyl amine and EDC. The molar ratios for EDC and methyl amine were in ten-fold excess of theoretical dendrimer terminal carboxylic acid groups. As a representative example: NADH-Gen 3.5 (3.85 µmols) was dissolved in pH 4.7, 100 mM MES buffer with EDC (0.468 g, 2.464 mmols) and stirred for 1 h at room temperature. Methyl amine (0.166 g, 2.464 mmols) was added to reaction and stirred overnight and purified using the same type of dialysis tubing as was used in the synthesis of NADH-GenXX dendrimers.

## Supplementary information

Supplementary Information

Description of Additional Supplementary Files

Supplementary Movie 1

## Data Availability

The authors declare that the data supporting the findings of this study are available within the paper and its Supplementary information files. Data are also available from the corresponding author upon reasonable request.

## References

[1] Tanaka S, Sawaya MR, Yeates TO (2010). Structure and mechanisms of a protein-based organelle in *Escherichia coli*. Science.

[2] Lin YC, Phua SC, Lin B, Inoue T (2013). Visualizing molecular diffusion through passive permeability barriers in cells: conventional and novel approaches. Curr. Opin. Chem. Biol..

[3] Lin YC (2013). Chemically inducible diffusion trap at cilia reveals molecular sieve-like barrier. Nat. Chem. Biol..

[4] Gora A, Brezovsky J, Damborsky J (2013). Gates of enzymes. Chem. Rev..

[5] Zhou H-X, Wlodek ST, McCammon JA (1998). Conformation gating as a mechanism for enzyme specificity. Proc. Natl Acad. Sci. USA.

[6] Wheeldon I (2016). Substrate channelling as an approach to cascade reactions. Nat. Chem..

[7] Estell D (1986). Probing steric and hydrophobic effects on enzyme-substrate interactions by protein engineering. Science.

[8] Sutormin OS, Sukovataya IE, Pande S, Kratasyuk VA (2018). Effect of viscosity on efficiency of enzyme catalysis of bacterial luciferase coupled with lactate dehydrogenase and NAD(P)H:FMN-Oxidoreductase. Mol. Catal..

[9] Yang X-Y (2017). Hierarchically porous materials: synthesis strategies and structure design. Chem. Soc. Rev..

[10] Gu C (2019). Design and control of gas diffusion process in a nanoporous soft crystal. Science.

[11] Zhou J, Wang B (2017). Emerging crystalline porous materials as a multifunctional platform for electrochemical energy storage. Chem. Soc. Rev..

[12] Yamashita H (2018). Single-site and nano-confined photocatalysts designed in porous materials for environmental uses and solar fuels. Chem. Soc. Rev..

[13] Timney BL (2016). Simple rules for passive diffusion through the nuclear pore complex. J. Cell Biol..

[14] Tanaka S (2008). Atomic-level models of the bacterial carboxysome shell. Science.

[15] Kwon S (2019). Structural basis of substrate recognition by a novel thermostable (S)-enantioselective omega-transaminase from *Thermomicrobium roseum*. Sci. Rep..

[16] Cannon GC, Heinhorst S, Kerfeld CA (2010). Carboxysomal carbonic anhydrases: structure and role in microbial CO2 fixation. Biochim. Biophys. Acta..

[17] Zhao Z (2016). Nanocaged enzymes with enhanced catalytic activity and increased stability against protease digestion. Nat. Commun..

[18] Slininger Lee MF, Jakobson CM, Tullman-Ercek D (2017). Evidence for improved encapsulated pathway behavior in a bacterial microcompartment through shell protein engineering. ACS Synth. Biol..

[19] Patterson DP, LaFrance B, Douglas T (2013). Rescuing recombinant proteins by sequestration into the P22 VLP. Chem. Commun. (Camb.).

[20] Roos W, Ivanovska I, Evilevitch A, Wuite G (2007). Viral capsids: mechanical characteristics, genome packaging and delivery mechanisms. Cell. Mol. Life Sci..

[21] Chakraborti S, Lin T-Y, Glatt S, Heddle JG (2020). Enzyme encapsulation by protein cages. RSC Adv..

[22] Roldao, A., Silva, A. C., Mellado, M. C. M., Alves, P. M. & Carrondo, M. J. T. Viruses and virus-like particles in biotechnology: fundamentals and applications. *Comprehensive biotechnology.* 625 (2011).

[23] Fiedler JD, Brown SD, Lau JL, Finn MG (2010). RNA-directed packaging of enzymes within virus-like particles. Angew. Chem. Int. Ed. Engl..

[24] Edwardson TGW, Hilvert D (2019). Virus-inspired function in engineered protein cages. J. Am. Chem. Soc..

[25] Waghwani, H. K. et al. Virus like particles (VLPs) as a platform for hierarchical compartmentalization. *Biomacromolecules* **26**, 2060–2072 (2020). 10.1021/acs.biomac.0c00030 32319761

[26] King J (1976). Structure and assembly of the capsid of bacteriophage P22. Philos. Trans. R. Soc. Lond. B, Biol. Sci..

[27] Weigele PR, Sampson L, Winn-Stapley D, Casjens SR (2005). Molecular genetics of bacteriophage P22 scaffolding protein’s functional domains. J. Mol. Biol..

[28] Parent KN (2010). P22 coat protein structures reveal a novel mechanism for capsid maturation: stability without auxiliary proteins or chemical crosslinks. Structure.

[29] Cortines JR, Motwani T, Vyas AA, Teschke CM (2014). Highly specific salt bridges govern bacteriophage P22 icosahedral capsid assembly: identification of the site in coat protein responsible for interaction with scaffolding protein. J. Virol..

[30] Chen DH (2011). Structural basis for scaffolding-mediated assembly and maturation of a dsDNA virus. Proc. Natl Acad. Sci. USA.

[31] O’Neil A, Reichhardt C, Johnson B, Prevelige PE, Douglas T (2011). Genetically programmed in vivo packaging of protein cargo and its controlled release from bacteriophage P22. Angew. Chem. Int Ed. Engl..

[32] Patterson DP, Prevelige PE, Douglas T (2012). Nanoreactors by programmed enzyme encapsulation inside the capsid of the bacteriophage P22. ACS Nano.

[33] Patterson, D. P. et al. Virus-like particle nanoreactors: programmed encapsulation of the thermostable CelB glycosidase inside the P22 capsid. *Soft Matter* **8**, 10.1039/c2sm26485d (2012).

[34] Jordan PC (2016). Self-assembling biomolecular catalysts for hydrogen production. Nat. Chem..

[35] McCoy K (2018). Cargo retention inside P22 virus-like particles. Biomacromolecules.

[36] Hryc CF (2017). Accurate model annotation of a near-atomic resolution cryo-EM map. Proc. Natl Acad. Sci. USA.

[37] Casjens S, King J (1974). P22 morphogenesis I: catalytic scaffolding protein in capsid assembly. J. Supramolecular Struct..

[38] Selivanovitch E, Koliyatt R, Douglas T (2019). Chemically induced morphogenesis of P22 virus-like particles by the surfactant sodium dodecyl sulfate. Biomacromolecules.

[39] Teschke CM, McGough A, Thuman-Commike PA (2003). Penton release from P22 heat-expanded capsids suggests importance of stabilizing penton-hexon interactions during capsid maturation. Biophysical J..

[40] Hadden, J. A. et al. All-atom molecular dynamics of the HBV capsid reveals insights into biological function and cryo-EM resolution limits. *Elife* **7**, 10.7554/eLife.32478 (2018). 10.7554/eLife.32478 PMC5927769 29708495

[41] Hadden JA, Perilla JR (2018). All-atom virus simulations. Curr. Opin. Virol..

[42] Zappelli P, Rossodivita A, RE L (1975). Synthesis of coenzymically active soluble and insoluble macromolecularized NAD+ derivatives. Eur. J. Biochem..

[43] Moriya T, Kawamata A, Takahashi Y, Iwabuchi Y, Kanoh N (2013). An improved fluorogenic NAD(P)+ detection method using 2-acetylbenzofuran: its origin and application. Chem. Commun. (Camb.).

[44] Lehninger, L. [126] Preparation of reduced DPN (chemical method). 885–887 (1957).

[45] Panza JL, Russell AJ, Beckman EJ (2002). Synthesis of fluorinated NAD as a soluble coenzyme for enzymatic chemistry in fluorous solvents and carbon dioxide. Tetrahedron.

[46] Lee C-Y, Kaplan NO (1975). Characteristics of 8-substituted adenine nucleotide derivatives utilized in affinity chromatography. Arch. Biochem. Biophys..

[47] Etrych T (2011). Biodegradable star HPMA polymer conjugates of doxorubicin for passive tumor targeting. Eur. J. Pharm. Sci..

[48] Pettersen EF (2004). UCSF Chimera—a visualization system for exploratory research and analysis. J. Computational Chem..

[49] Smart OS, Neduvelil JG, Wang X, Wallace B, Sansom MS (1996). HOLE: a program for the analysis of the pore dimensions of ion channel structural models. J. Mol. Graph..

[50] Renkin EM (1954). Filtration, diffusion, and molecular sieving through porous cellulose membranes. J. Gen. Physiol..

[51] Bassingthwaighte JB (2006). A practical extension of hydrodynamic theory of porous transport for hydrophilic solutes. Microcirculation.

[52] Yifrach O (2004). Hill coefficient for estimating the magnitude of cooperativity in gating transitions of voltage-dependent ion channels. Biophys. J..

[53] Uchida M (2018). Modular self-assembly of protein cage lattices for multistep catalysis. ACS Nano.

[54] McCoy K, Uchida M, Lee B, Douglas T (2018). Templated assembly of a functional ordered protein macromolecular framework from P22 Virus-like particles. ACS Nano.

[55] Niu Y, Sun L, Crooks RM (2003). Determination of the intrinsic proton binding constants for poly (amidoamine) dendrimers via potentiometric pH titration. Macromolecules.

[56] Glasgow JE, Asensio MA, Jakobson CM, Francis MB, Tullman-Ercek D (2015). Influence of electrostatics on small molecule flux through a protein nanoreactor. ACS Synth. Biol..

[57] Azuma Y, Bader DLV, Hilvert D (2018). Substrate sorting by a supercharged nanoreactor. J. Am. Chem. Soc..

[58] Ripoll DR, Faerman CH, Axelsen PH, Silman I, Sussman JL (1993). An electrostatic mechanism for substrate guidance down the aromatic gorge of acetylcholinesterase. Proc. Natl Acad. Sci. USA.

[59] Botti SA, Felder CE, Sussman JL, Silman I (1998). Electrotactins: a class of adhesion proteins with conserved electrostatic and structural motifs. Protein Eng..

[60] Yang X, Chasteen ND (1996). Molecular diffusion into horse spleen ferritin: a nitroxide radical spin probe study. Biophysical J..

[61] Porschke D (1996). Electrooptical measurements demonstrate a large permanent dipole moment associated with acetylcholinesterase. Biophysical J..

[62] Douglas T, Ripoll DR (1998). Calculated electrostatic gradients in recombinant human H‐chain ferritin. Protein Sci..

[63] Sankararamakrishnan R, Adcock C, Sansom M (1996). The pore domain of the nicotinic acetylcholine receptor: molecular modeling, pore dimensions, and electrostatics. Biophysical J..

