# Supplementary material for: Molecular exclusion limits for diffusion across a porous capsid
Source: Nat Commun. 2021 May 18;12:2903. doi: 10.1038/s41467-021-23200-1 (PMC8131759; doi:10.1038/s41467-021-23200-1)
Supplement: Supplementary file 1 — Supplementary Information [file 41467_2021_23200_MOESM1_ESM.pdf]

Supplementary Information

Molecular Exclusion Limits for Diffusion Across a Porous Capsid

Ekaterina Selivanovitch[1], Benjamin LaFrance[2], Trevor Douglas[1*]

[1] Department of Chemistry, Indiana University, Bloomington, IN 47405

[2] Department of Molecular and Cell Biology, University of California Berkeley, Berkeley, CA 94720

Sequences

Coat Protein DNA and protein sequences

ATGGCTTTGAACGAAGGTCAAATTGTTACACTGGCGGTAGATGAAATC
ATCGAAACCATCTCCGCAATCACTCCAATGGCGCAGAAAGCCAAGAA
ATACACCCCGCCTGCTGCTTCTATGCAGCGCTCCAGCAATACCATCTG
GATGCCTGTAGAGCAAGAGTCACCCACTCAGGAGGGCTGGGATTTAA
CTGATAAAGCGACAGGGTTACTGGAACTTAACGTCGCGGTAAACATG
GGAGAGCCGGATAACGACTTCTTCCAGTTGCGTGCTGATGACTTGCGA
GACGAAACTGCGTATCGTCGCCGCATCCAGTCTGCCGCTCGCAAGCTG
GCGAACAACGTTGAGTTGAAAGTCGCAAACATGGCCGCCGAGATGGG
TTCGCTGGTTATCACCTCCCCTGATGCCATCGGCACTAATACCGCAGA
CGCCTGGAACTTTGTGGCCGACGCAGAAGAAATCATGTTCTCCCGCGA
ACTTAACCGCGACATGGGGACATCGTACTTCTTCAACCCTCAGGACTA
CAAAAAAGCGGGTTACGACCTGACCAAGCGTGACATCTTCGGGCGTAT
TCCTGAAGAAGCATACCGAGATGGCACCATTCAGCGTCAGGTCGCTGG
CTTCGATGATGTCCTGCGCTCTCCGAAACTTCCTGTGCTGACCAAATCC
ACCGCAACTGGCATCACTGTATCCGGTGCGCAGTCCTTCAAGCCTGTC
GCATGGCAACTGGATAACGATGGCAACAAAGTTAACGTTGATAACCG
TTTTGCTACCGTCACCCTGTCTGCAACTACCGGCATGAAACGCGGCGA
CAAAATTTCGTTTGCTGGCGTTAAGTTCCTTGGTCAGATGGCTAAGAA
CGTACTGGCTCAGGATGCGACTTTCTCCGTAGTCCGCGTTGTTGACGGT
ACTCATGTTGAAATCACGCCGAAGCCGGTAGCGCTGGATGATGTTTCC
CTGTCTCCGGAGCAGCGTGCCTACGCCAACGTTAACACCTCGCTGGCT
GATGCAATGGCAGTGAACATTCTGAACGTTAAAGACGCTCGCACTAAT
GTGTTCTGGGCTGACGATGCTATTCGTATCGTGTCTCAGCCGATTCCGG
CTAACCATGAACTTTTTGCAGGTATGAAAACTACCTCATTCAGCATCC
CTGATGTTGGCCTGAACGGTATCTTCGCTACGCAGGGTGATATTTCCA
CCCTGTCCGGCCTGTGCCGTATTGCGCTGTGGTACGGCGTAAACGCGA
CACGACCGGAGGCAATCGGTGTTGGCCTGCCTGGTCAGACTGCGTAAT
AG

MALNEGQIVTLAVDEIIETISAITPMAQKAKKYTPPAASMQRSSNTIWMPV
EQESPTQEGWDLTDKATGLLELNVAVNMGEPDNDFFQLRADDLRDETA
YRRRIQSAARKLANNVELKVANMAAEMGSLVITSPDAIGTNTADAWNFV
ADAEEIMFSRELNRDMGTSYFFNPQDYKKAGYDLTKRDIFGRIPEEAYRD
GTIQRQVAGFDDVLRSPKLPVLTKSTATGITVSGAQSFKPVAWQLDNDGN
KVNVDNRFATVTLSATTGMKRGDKISFAGVKFLGQMAKNVLAQDATFS
VVRVVDGTHVEITPKPVALDDVSLSPEQRAYANVNTSLADAMAVNILNV
KDARTNVFWADDAIRIVSQPIPANHELFAGMKTTSFSIPDVGLNGIFATQG
DISTLSGLCRIALWYGVNATRPEAIGVGLPGQTA

Scaffold Protein DNA and protein sequences (AdhD-SP)

ATGGGCAGCTCGCACCATCATCACCATCACAGCGGCGCAAAACGTGTC
AATGCTTTTAACGACCTGAAGCGCATCGGCGACGACAAGGTTACCGCT
ATCGGGATGGGAACCTGGGGAATCGGCGGACGTGAGACGCCAGATTA
TAGTCGTGACAAGGAAAGTATCGAGGCCATCCGCTATGGTCTGGAGCT
GGGCATGAATTTGATTGACACGGCTGAATTTTACGGGGCAGGTCATGC
CGAAGAAATTGTTGGAGAGGCAATTAAAGAGTTTGAGCGTGAAGATA
TTTTCATCGTTAGTAAGGTTTGGCCAACCCACTTTGGCTACGAGGAAG
CGAAGAAGGCCGCGCGCGCGTCGGCAAAGCGTTTGGGAACATACATT
GACTTGTACCTTCTTCACTGGCCTGTCGATGATTTTAAGAAAATCGAA
GAAACTCTTCATGCTCTTGAAGATTTGGTTGACGAGGGTGTCATTCGTT
ACATCGGAGTCAGCAATTTTAACCTGGAACTTCTTCAACGTTCACAGG
AAGTTATGCGTAAGTACGAAATCGTTGCAAACCAAGTAAAGTATTCCG
TAAAGGACCGCTGGCCCGAAACGACGGGACTTTTAGATTACATGAAG
CGTGAGGGCATTGCCTTGATGGCTTATACTCCTTTAGAGAAGGGTACG
TTAGCTCGTAATGAATGCTTGGCCAAAATCGGAGAAAAGTATGGAAA
GACCGCAGCTCAGGTCGCTTTGAACTACCTTATCTGGGAGGAGAATGT
CGTGGCAATCCCTAAGGCATCAAATAAGGAGCACTTAAAAGAGAACT
TTGGGGCCATGGGGTGGCGTCTTTCAGAAGAGGATCGTGAAATGGCTC
GCCGCTGCGTGGGTGCAGCAGGTGAAAACCTGTATTTCCAGAGCGGTG
CGGCAGGCCGCAGCAATGCCGTAGCAGAACAGGGCCGCAAGACTCAG
GAGTTTACCCAGCAATCAGCGCAATACGTCGAAGCTGCCCGCAAACA
CTATGACGCGGCGGAAAAGCTCAACATCCCTGACTATCAGGAGAAAG
AAGACGCATTTATGCAACTGGTTCCGCCTGCGGTTGGGGCCGACATTA
TGCGCCTGTTCCCGGAAAAGTCCGCCGCGCTCATGTATCACCTGGGGG
CAAACCCGGAGAAAGCCCGCCAGTTACTGGCGATGGATGGGCAGTCC
GCGCTGATTGAACTCACTCGACTATCCGAACGCTTAACTCTCAAGCCT
CGCGGTAAACAAATCTCTTCCGCTCCCCATGCTGACCAGCCTATTACC
GGTGATGTCAGCGCAGCAAATAAAGATGCCATTCGTAAACAAATGGA
TGCTGCTGCGAGCAAGGGAGATGTGGAAACCTACCGCAAGCTAAAGG
CAAAACTTAAAGGAATCCGATAA

MGSSHHHHHHSGAKRVNAFNDLKRIGDDKVTAIGMGTWGIGGRETPDY
SRDKESIEAIRYGLELGMNLIDTAEFYGAGHAEEIVGEAIKEFEREDIFIVSK
VWPTHFGYEEAKKAARASAKRLGTYIDLYLLHWPVDDFKKIEETLHALE
DLVDEGVIRYIGVSNFNLELLQRSQEVMRKYEIVANQVKYSVKDRWPET
TGLLDYMKREGIALMAYTPLEKGTLARNECLAKIGEKYGKTAAQVALNY
LIWEENVVAIPKASNKEHLKENFGAMGWRLSEEDREMARRCVGAAGEN
LYFQSGAAGRSNAVAEQGRKTQEFTQQSAQYVEAARKHYDAAEKLNIPD
YQEKEDAFMQLVPPAVGADIMRLFPEKSAALMYHLGANPEKARQLLAM
DGQSALIELTRLSERLTLKPRGKQISSAPHADQPITGDVSAANKDAIRKQM
DAAASKGDVETYRKLKAKLKGIRStop

Coat Protein K184Q/R203S DNA and protein sequences

ATGGCTTTGAACGAAGGTCAAATTGTTACACTGGCGGTAGATGAAATC
ATCGAAACCATCTCCGCAATCACTCCAATGGCGCAGAAAGCCAAGAA
ATACACCCCGCCTGCTGCTTCTATGCAGCGCTCCAGCAATACCATCTG

GATGCCTGTAGAGCAAGAGTCACCCACTCAGGAGGGCTGGGATTTAA
CTGATAAAGCGACAGGGTTACTGGAACTTAACGTCGCGGTAAACATG
GGAGAGCCGGATAACGACTTCTTCCAGTTGCGTGCTGATGACTTGCGA
GACGAAACTGCGTATCGTCGCCGCATCCAGTCTGCCGCTCGCAAGCTG
GCGAACAACGTTGAGTTGAAAGTCGCAAACATGGCCGCCGAGATGGG
TTCGCTGGTTATCACCTCCCCTGATGCCATCGGCACTAATACCGCAGA
CGCCTGGAACTTTGTGGCCGACGCAGAAGAAATCATGTTCTCCCGCGA
ACTTAACCGCGACATGGGGACATCGTACTTCTTCAACCCTCAGGACTA
CAAAAAAGCGGGTTACGACCTGACCCAGCGTGACATCTTCGGGCGTAT
TCCTGAAGAAGCATACCGAGATGGCACCATTCAGAGTCAGGTCGCTGG
CTTCGATGATGTCCTGCGCTCTCCGAAACTTCCTGTGCTGACCAAATCC
ACCGCAACTGGCATCACTGTATCCGGTGCGCAGTCCTTCAAGCCTGTC
GCATGGCAACTGGATAACGATGGCAACAAAGTTAACGTTGATAACCG
TTTTGCTACCGTCACCCTGTCTGCAACTACCGGCATGAAACGCGGCGA
CAAAATTTCGTTTGCTGGCGTTAAGTTCCTTGGTCAGATGGCTAAGAA
CGTACTGGCTCAGGATGCGACTTTCTCCGTAGTCCGCGTTGTTGACGGT
ACTCATGTTGAAATCACGCCGAAGCCGGTAGCGCTGGATGATGTTTCC
CTGTCTCCGGAGCAGCGTGCCTACGCCAACGTTAACACCTCGCTGGCT
GATGCAATGGCAGTGAACATTCTGAACGTTAAAGACGCTCGCACTAAT
GTGTTCTGGGCTAACGATGCTATTCGTATCGTGTCTCAGCCGATTCCGG
CTAACCATGAACTTTTTGCAGGTATGAAAACTACCTCATTCAGCATCC
CTGATGTTGGCCTGAACGGTATCTTCGCTACGCAGGGTGATATTTCCA
CCCTGTCCGGCCTGTGCCGTATTGCGCTGTGGTACGGCGTAAACGCGA
CACGACCGGAGGCAATCGGTGTTGGCCTGCCTGGTCAGACTGCGTAAT
AG

MALNEGQIVTLAVDEIIETISAITPMAQKAKKYTPPAASMQRSSNTIWMPV
EQESPTQEGWDLTDKATGLLELNVAVNMGEPDNDFFQLRADDLRDETA
YRRRIQSAARKLANNVELKVANMAAEMGSLVITSPDAIGTNTADAWNFV
ADAEEIMFSRELNRDMGTSYFFNPQDYKKAGYDLTQRDIFGRIPEEAYRD
GTIQSQVAGFDDVLRSPKLPVLTKSTATGITVSGAQSFKPVAWQLDNDGN
KVNVDNRFATVTLSATTGMKRGDKISFAGVKFLGQMAKNVLAQDATFS
VVRVVDGTHVEITPKPVALDDVSLSPEQRAYANVNTSLADAMAVNILNV
KDARTNVFWADDAIRIVSQPIPANHELFAGMKTTSFSIPDVGLNGIFATQG
DISTLSGLCRIALWYGVNATRPEAIGVGLPGQTAStop

Coat Protein D143N/D357N DNA and protein sequences

ATGGCTTTGAACGAAGGTCAAATTGTTACACTGGCGGTAGATGAAATC
ATCGAAACCATCTCCGCAATCACTCCAATGGCGCAGAAAGCCAAGAA
ATACACCCCGCCTGCTGCTTCTATGCAGCGCTCCAGCAATACCATCTG
GATGCCTGTAGAGCAAGAGTCACCCACTCAGGAGGGCTGGGATTTAA
CTGATAAAGCGACAGGGTTACTGGAACTTAACGTCGCGGTAAACATG
GGAGAGCCGGATAACGACTTCTTCCAGTTGCGTGCTGATGACTTGCGA

GACGAAACTGCGTATCGTCGCCGCATCCAGTCTGCCGCTCGCAAGCTG
GCGAACAACGTTGAGTTGAAAGTCGCAAACATGGCCGCCGAGATGGG
TTCGCTGGTTATCACCTCCCCTGATGCCATCGGCACTCATACCGCAAA
CGCCTGGAACTTTGTGGCCGACGCAGAAGAAATCATGTTCTCCCGCGA
ACTTAACCGCGACATGGGGACATCGTACTTCTTCAACCCTCAGGACTA
CAAAAAAGCGGGTTACGACCTGACCAAGCGTGACATCTTCGGGCGTAT
TCCTGAAGAAGCATACCGAGATGGCACCATTCAGCGTCAGGTCGCTGG
CTTCGATGATGTCCTGCGCTCTCCGAAACTTCCTGTGCTGACCAAATCC
ACCGCAACTGGCATCACTGTATCCGGTGCGCAGTCCTTCAAGCCTGTC
GCATGGCAACTGGATAACGATGGCAACAAAGTTAACGTTGATAACCG
TTTTGCTACCGTCACCCTGTCTGCAACTACCGGCATGAAACGCGGCGA
CAAAATTTCGTTTGCTGGCGTTAAGTTCCTTGGTCAGATGGCTAAGAA
CGTACTGGCTCAGGATGCGACTTTCTCCGTAGTCCGCGTTGTTGACGGT
ACTCATGTTGAAATCACGCCGAAGCCGGTAGCGCTGGATGATGTTTCC
CTGTCTCCGGAGCAGCGTGCCTACGCCAACGTTAACACCTCGCTGGCT
GATGCAATGGCAGTGAACATTCTGAACGTTAAAGACGCTCGCACTAAT
GTGTTCTGGGCTGACAATGCTATTCGTATCGTGTCTCAGCCGATTCCGG
CTAACCATGAACTTTTTGCAGGTATGAAAACTACCTCATTCAGCATCC
CTGATGTTGGCCTGAACGGTATCTTCGCTACGCAGGGTGATATTTCCA
CCCTGTCCGGCCTGTGCCGTATTGCGCTGTGGTACGGCGTAAACGCGA
CACGACCGGAGGCAATCGGTGTTGGCCTGCCTGGTCAGACTGCGTAAT
AG

MALNEGQIVTLAVDEIIETISAITPMAQKAKKYTPPAASMQRSSNTIWMPV
EQESPTQEGWDLTDKATGLLELNVAVNMGEPDNDFFQLRADDLRDETA
YRRRIQSAARKLANNVELKVANMAAEMGSLVITSPDAIGTHTANAWNFV
ADAEEIMFSRELNRDMGTSYFFNPQDYKKAGYDLTKRDIFGRIPEEAYRD
GTIQRQVAGFDDVLRSPKLPVLTKSTATGITVSGAQSFKPVAWQLDNDGN
KVNVDNRFATVTLSATTGMKRGDKISFAGVKFLGQMAKNVLAQDATFS
VVRVVDGTHVEITPKPVALDDVSLSPEQRAYANVNTSLADAMAVNILNV
KDARTNVFWADNAIRIVSPIPANHELFAGMKTTSFSIPDVGLNGIFATQGD
ISTLSGLCRIALWYGVNATRPEAIGVGLPGQTAStop

** A random N140H mutation was inserted during the cloning process . This was confirmed using sequencing and mass spectroscopy analysis.

Supplementary Table 1: List of DNA Primers

Mutants	Forward
D143N	5' GGCACTAATACCGCAAACGCCTGGAACTTTG 3'
K184Q	5' GTTACGACCTGACCCAGCGTGACATCTTC 3'
D357N	5' GTGTTCTGGGCTGACAATGCTATTCGTATC 3'
R203S	5' GGCACCATTCAGAGTCAGGTCGCTG 3'
	Reverse
D143N	5' CAAAGTTCCAGGCGTTTGCGGTTTAGTGCC 3'
K184Q	5' GAAGATGTCACGCTGGGTCAGGTCGTAAC 3'
D357N	5' GATACGAATAGCATTGTCAGCCCAGAACAC 3'
R203S	5' CAGCGACCTGACTCTGAATGGTGCC 3'

Supplementary Figures

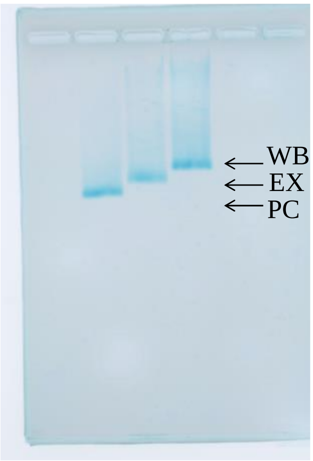

Supplementary Fig.1
Native agarose gel with P22 procapsid (PC), Expanded (EX),and Wiffleball (WB) morphologies. Each lane shows one band corresponding to its respective morphology.

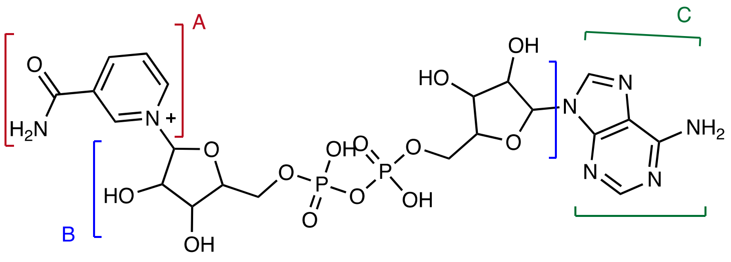

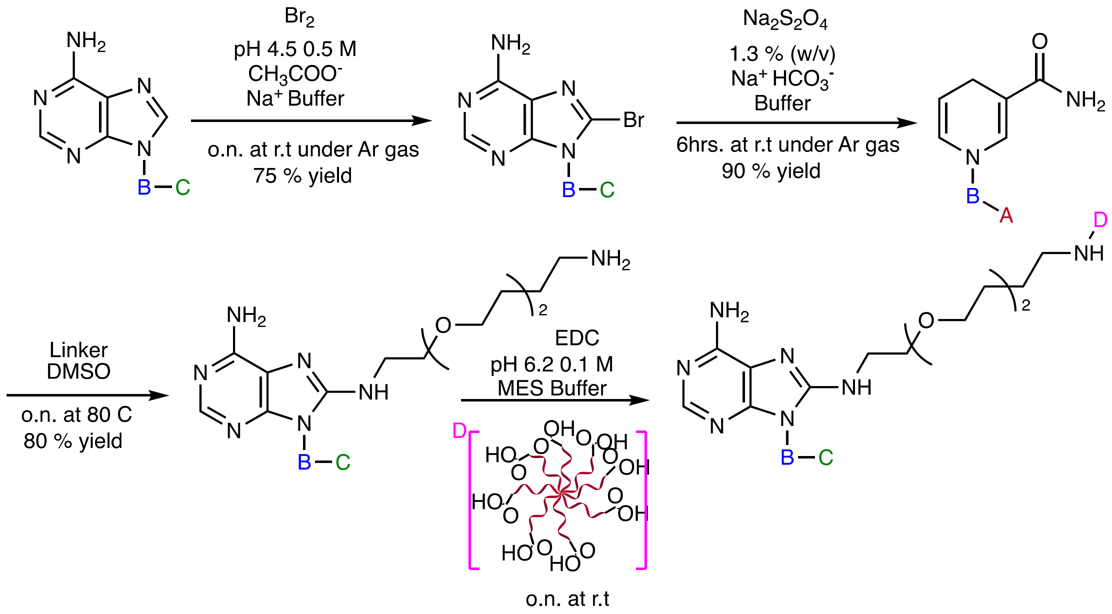

Supplementary Fig.2

Schematic of NADH-Neg$_{xx}$ conjugate synthesis. The details for each step can be found in the Methods section.

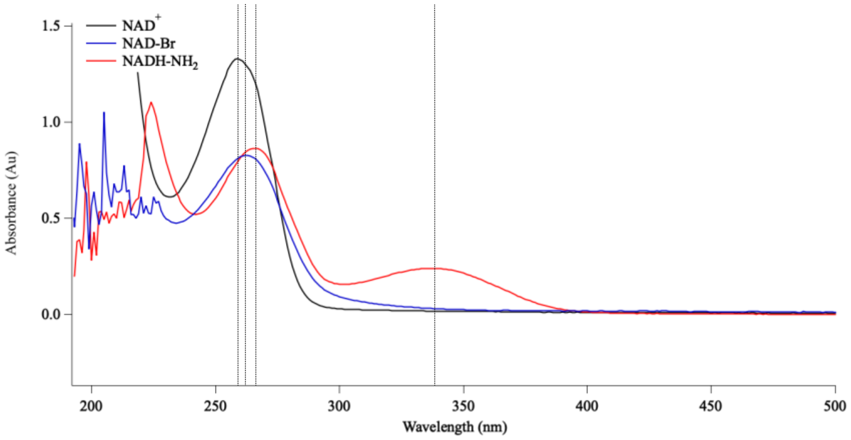

Supplementary Fig.3
UV-VIS spectra of NAD^+, NAD^+-Br, NADH-NH_2 showing a shift in λ_{max} after $NAD^+ \rightarrow NAD^+$Br chemical transformation, and the appearance of peak at 340 nm upon reduction reaction (NAD^+-Br$\rightarrow$NADH-Br) and another red shift after NADH-Br$\rightarrow$NADH-NH_2.

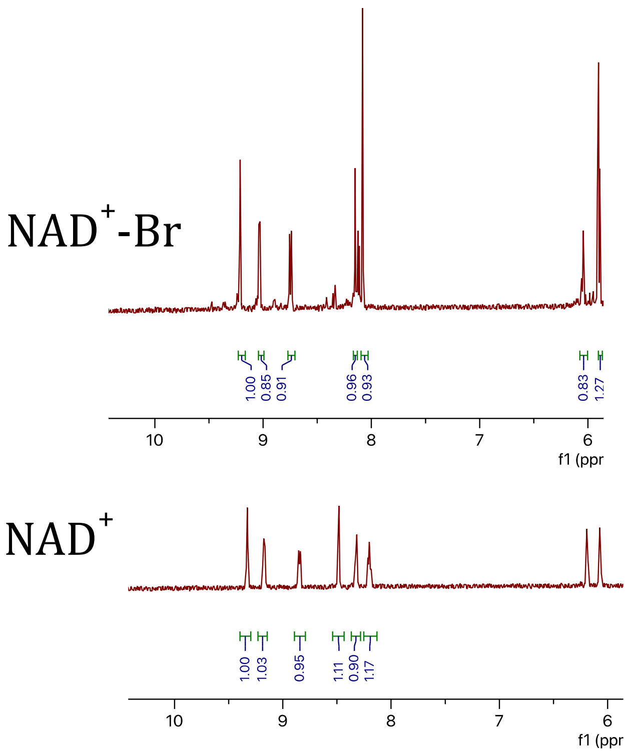

Supplementary Fig.4
NMR Spectra of Sigma Aldrich β-Nicotinamide adenine dinucleotide (NAD^+) and modified NAD^+Br showing the aromatic region pertaining to the protons found on adenine and nicotinamide. NAD^+Br has 5 total aromatic protons due to the substitution of the C8 proton with bromine. (Full spectrum can be found in Supplementary Fig. 34)

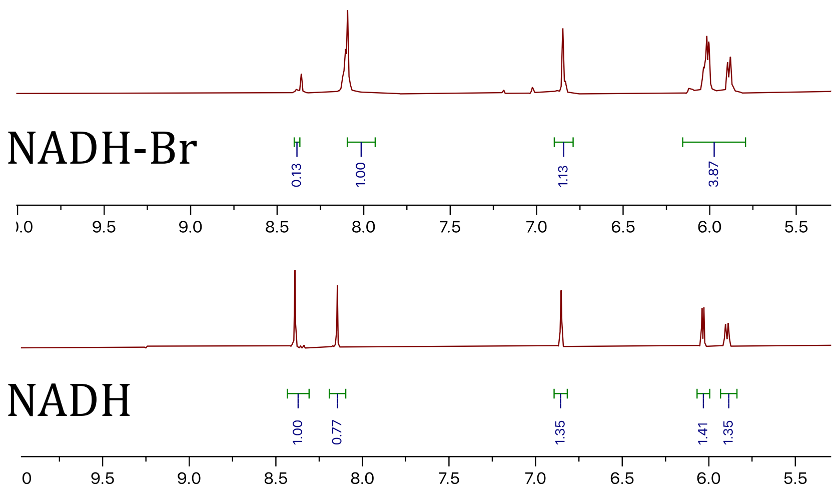

Supplementary Fig.5
NMR Spectra of Sigma Aldrich β-Nicotinamide adenine dinucleotide reduced form (NADH) and modified NADH-Br showing the aromatic region pertaining to the protons found on adenine and nicotinamide. Since the nicotinamide is no longer aromatic, protons have shifted upfield. The NADH adenine contains 2 protons, while the modified NADH-Br contains 1 proton and small impurity peak. (Full spectrum can be found in Supplementary Fig. 35)

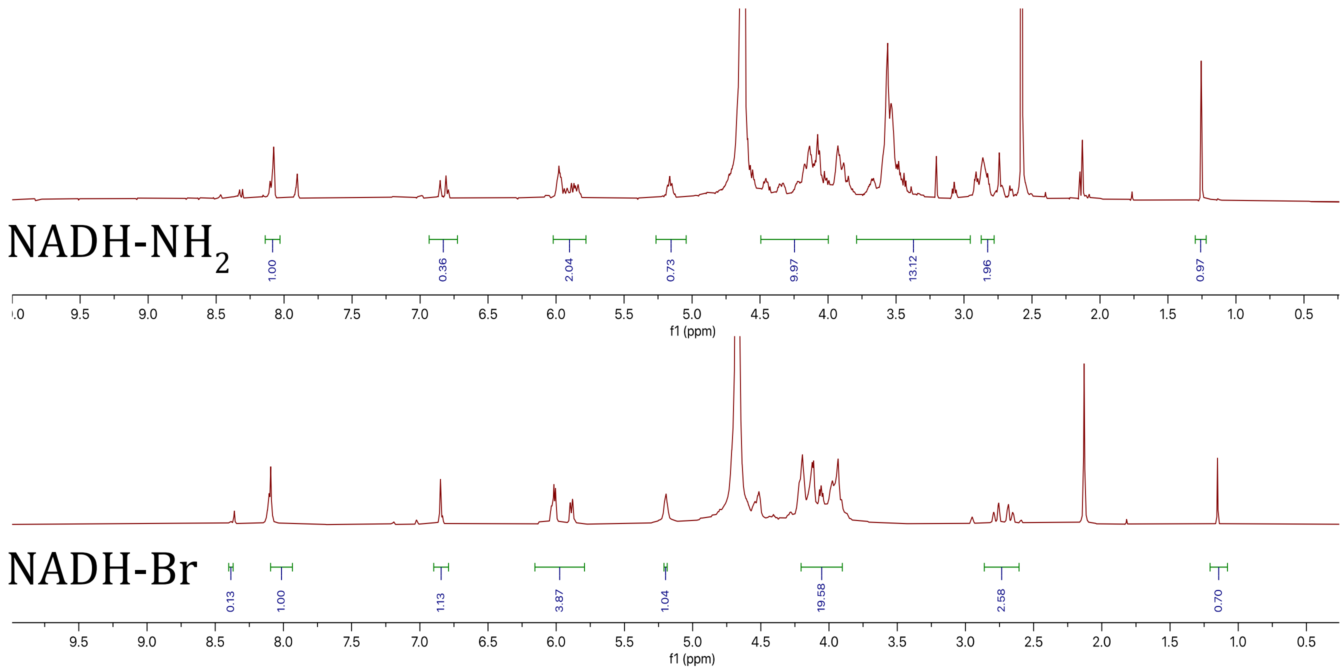

Supplementary Fig.6

NMR Spectra of NADH-Br and NADH-NH_2 showing additional upfield peaks resulting from the protons found on the linker. There are slight impurities seen from the water, acetate buffer, and acetone. (Full spectrum can be found in Supplementary Fig. 36)

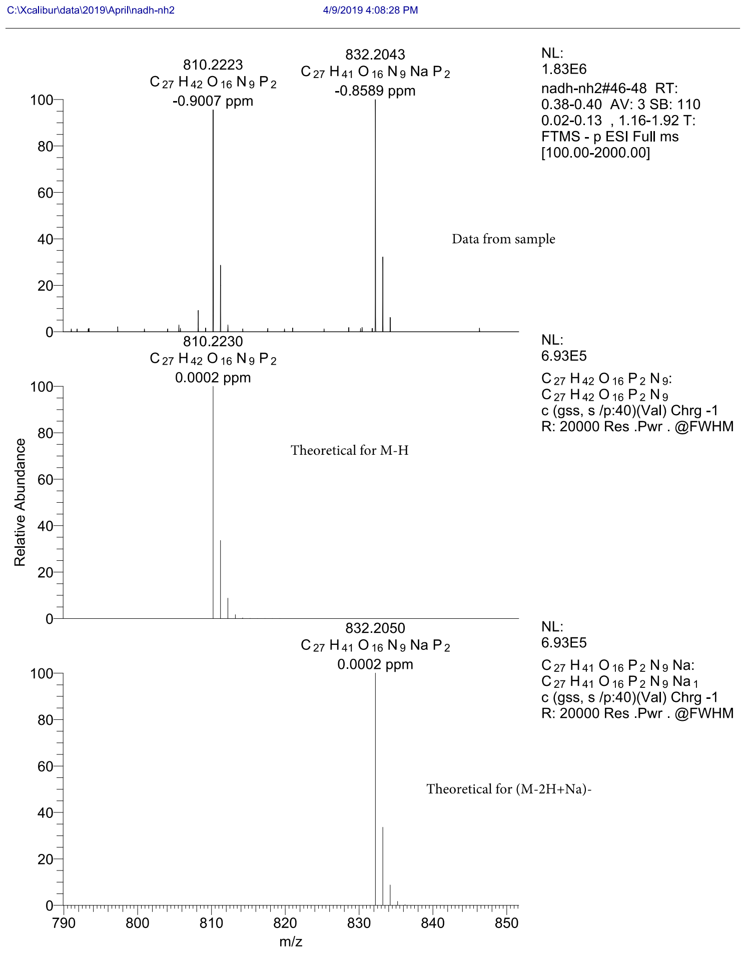

Supplementary Fig.7

MS data collected on the NADH-NH_2 with an observed molecular weight of 810 g/mol (theoretical: 810g/mol). The observed MW of 832 g/mol is due to the detection of a sodium cation, which would serve as a counterion for the phosphate backbone.

Dendrimer Characterization

Generation	D_h Dendrimer(nm)	Std Dev.
1.5	2.01	±0.08
2.5	3.16	±0.10
3.5	4.37	±0.06
4.5	5.56	±0.24
5.5	6.95	±0.04

Generation		
0.5	1.26	±0.20
1.5	2.53	±0.3
2.5	3.39	±0.06
3.5	4.33	±0.02
4.5	6.44	±0.06
5.5	7.79	±0.06

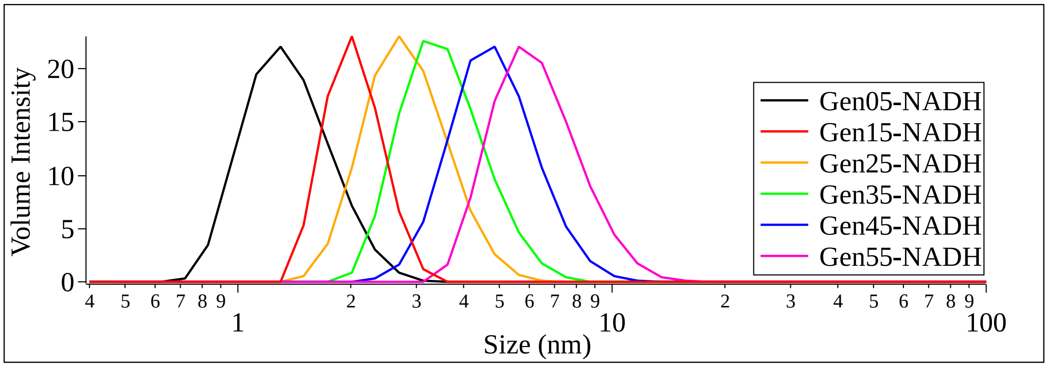

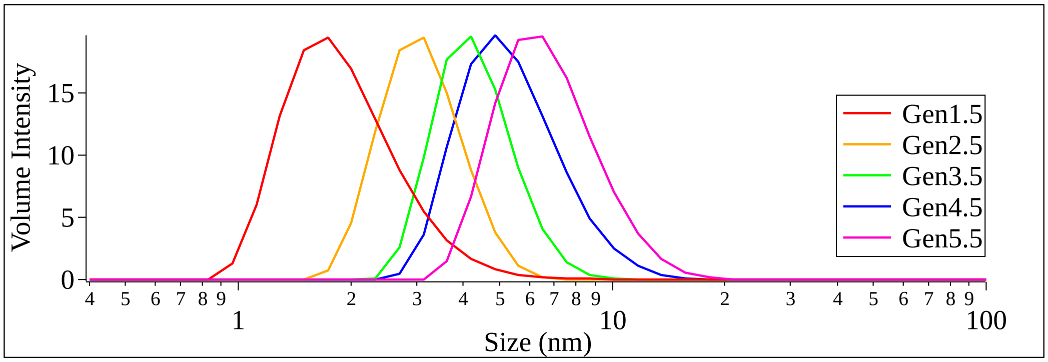

Supplementary Fig.8
DLS measurements comparing stock Sigma Aldrich PAMAM dendrimers and Dendrimer-NADH conjugates. Data for stock PAMAM Generation 0.5 could not be obtained due to large error range. D_h = Hydrodynamic Diameter

Generation Dendrimer-NADH	Zetapotential(mV)	StdDev
0.5	-9.56	±0.32
1.5	-11.4	±0.771
2.5	-11	±0.571
3.5	-12	±0.377
4.5	-16.2	±0.784
5.5	-18.2	±0.648

Supplementary Fig.9
Zeta potential measurements obtained for Dendrimer-NADH conjugates.

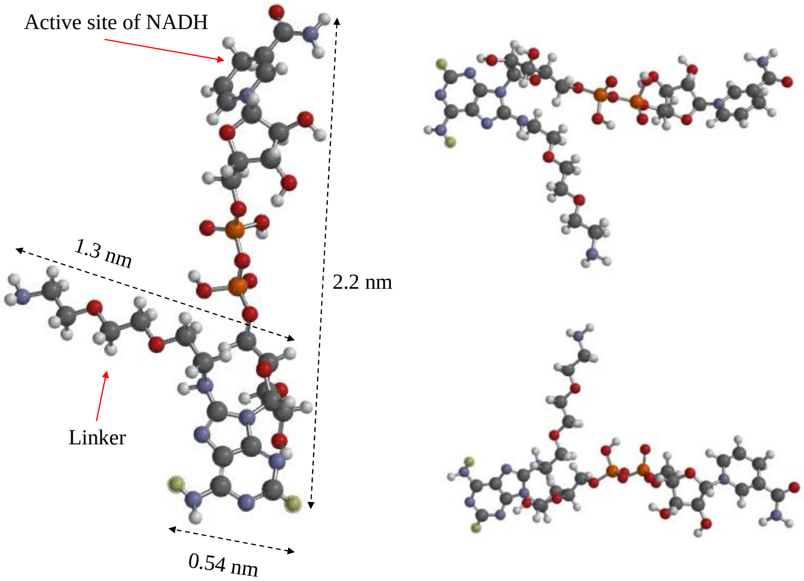

Supplementary Fig.10

Model of NADH molecule with linker and it’s theoretical dimensions in its thermodynamically most stable conformation. Image and energy minimization were obtained using Spartan software. Red sphere= oxygen, blue= nitrogen, grey= carbon, white= hydrogen, yellow highlight= a representative example of the atom selected for identifying dimensions.

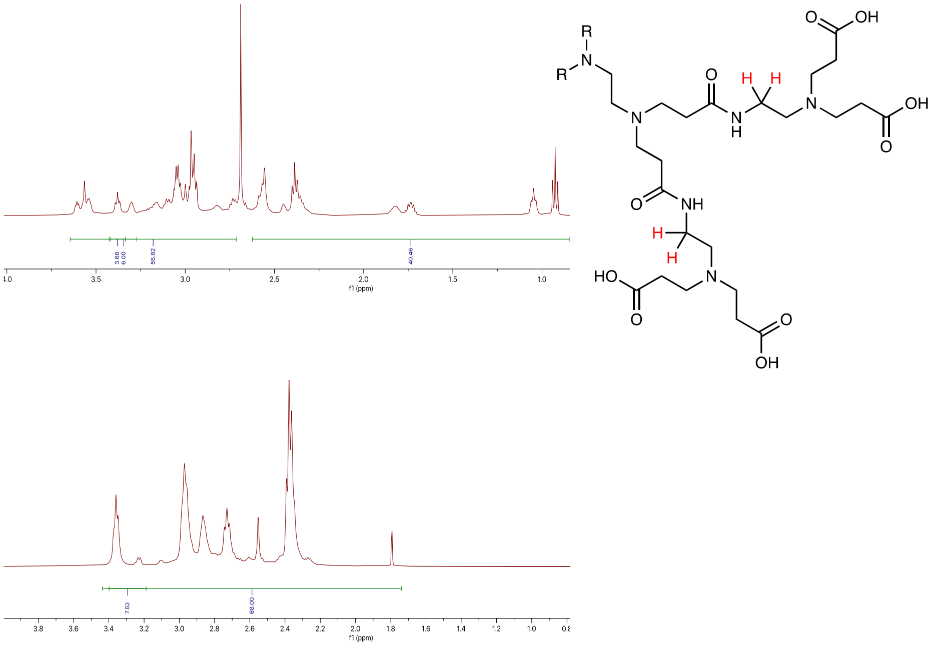

Supplementary Fig.11
NMR spectra of stock PAMAM Dendrimer Generation 0.5 (bottom) and NADH-Neg$_{0.5}$. The highlighted protons have been identified to be vicinal to the amide nitrogen. Those same protons were then identified on the NADH-Neg$_{0.5}$ spectrum. Using those peaks as a reference, assuming 1:1 stoichiometry of NADH-NH_2 to Gen0.5 Dendrimer, a total of 106 protons were found. This would indicate that there was an average of one NADH-NH_2 molecule per dendrimer. The peak at 2.7 was a solvent impurity peak from DMSO. (Full spectrum can be found in Supplementary Fig. 37)

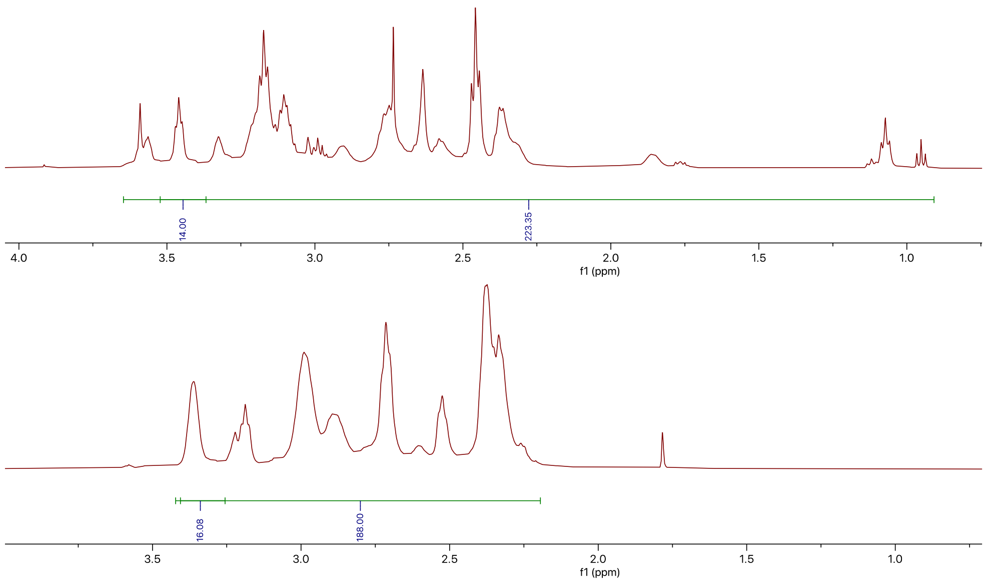

Supplementary Fig.12
NMR spectra of stock PAMAM Dendrimer Generation 1.5 (bottom) and NADH-Neg$_{1.5}$. The peaks pertaining to the protons vicinal to the amide were identified on the NADH-Neg$_{1.5}$ spectrum. Using those peaks as a reference, assuming 1:1 stoichiometry of NADH-NH_2 to Gen1.5 Dendrimer, a total of 223 protons were found. This would indicate that there was an average of one NADH-NH_2 molecule per dendrimer. The solvent impurity from DMSO is still in the spectrum, but other peaks are adjacent to it, therefore it was included in integrations of all Dendrimer-NADH conjugates from Gen1.5-5.5. (Full spectrum can be found in Supplementary Fig. 38)

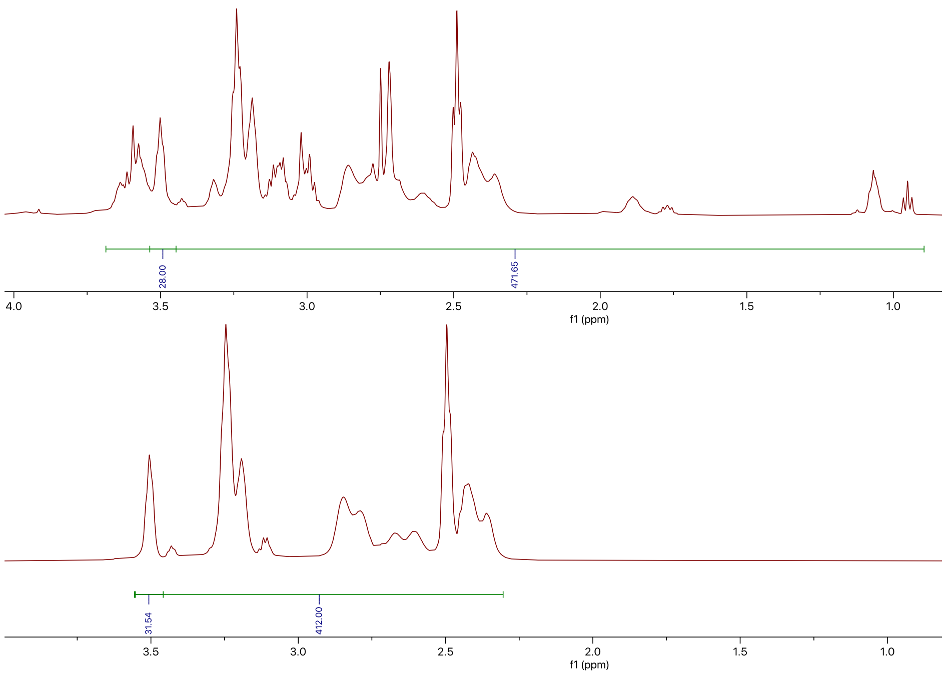

Supplementary Fig.13

NMR spectra of stock PAMAM Dendrimer Generation 2.5 (bottom) and NADH-Neg$_{2.5}$. The peaks pertaining to the protons vicinal to the amide were identified on the NADH-Neg$_{2.5}$ spectrum. Using those peaks as a reference, assuming 2:1 stoichiometry of NADH-NH_2 to Gen1.5 Dendrimer, a total of 472 protons were found. This would indicate that there was an average of two NADH-NH_2 molecules per dendrimer.

(Full spectrum can be found in Supplementary Fig. 39)

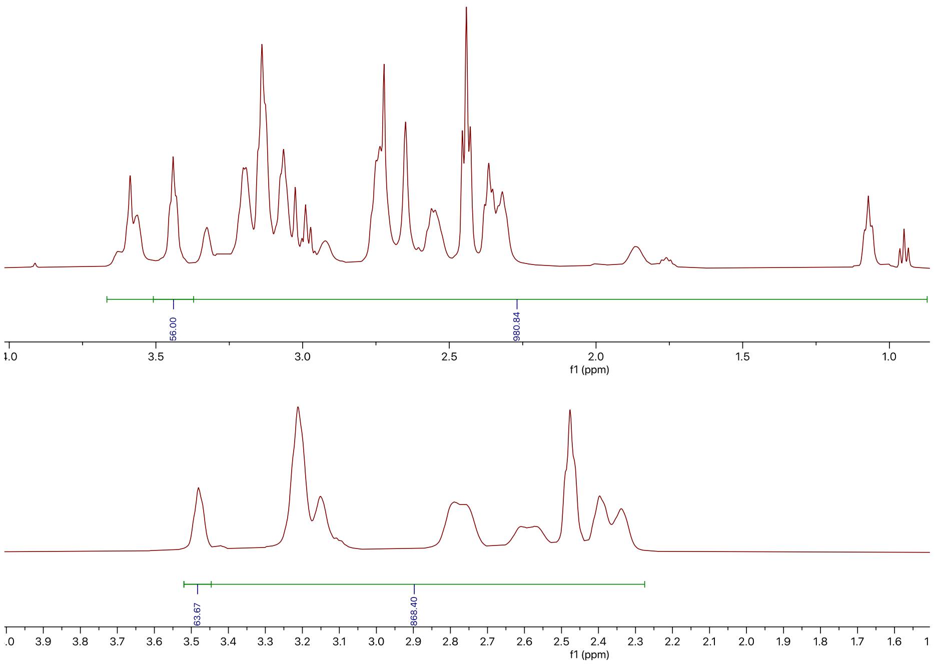

Supplementary Fig.14

NMR spectra of stock PAMAM Dendrimer Generation 3.5 (bottom) and NADH-Neg$_{3.5}$. The peaks pertaining to the protons vicinal to the amide were identified on the NADH-Neg$_{3.5}$ spectrum. Using those peaks as a reference, assuming 8:1 stoichiometry of NADH-NH$_2$ to Gen3.5 Dendrimer, a total of 960 protons were found. This would indicate that there was an average of four NADH-NH$_2$ molecules per dendrimer.

(Full spectrum can be found in Supplementary Fig. 40)

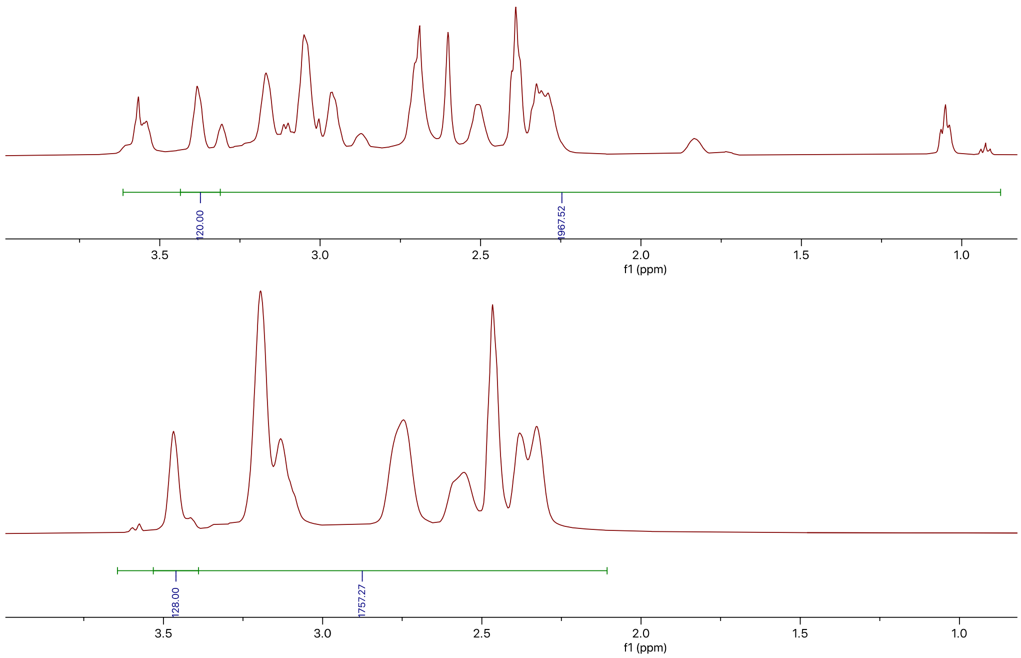

Supplementary Fig.15

NMR spectra of stock PAMAM Dendrimer Generation4.5 (bottom) and NADH-Neg$_{4.5}$. The peaks pertaining to the protons vicinal to the amide were identified on the NADH-Neg$_{4.5}$ spectrum. Using those peaks as a reference, assuming 16:1 stoichiometry of NADH-NH_2 to Gen3.5 Dendrimer, a total of 1,967 protons were found. This would indicate that there was an average of eight NADH-NH_2 molecules per dendrimer. (Full spectrum can be found in Supplementary Fig. 41)

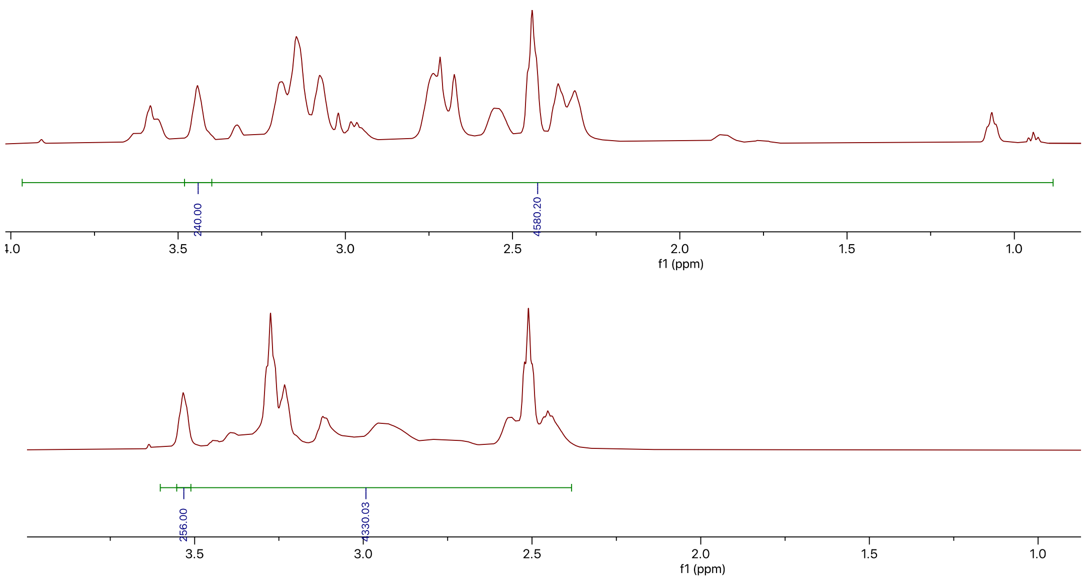

Supplementary Fig.16
NMR spectra of stock PAMAM Dendrimer Generation 5.5 (bottom) and NADH-Neg$_{5.5}$. The peaks pertaining to the protons vicinal to the amide were identified on the NADH-Neg$_{5.5}$ spectrum. Using those peaks as a reference, assuming 16:1 stoichiometry of NADH-NH_2 to Gen5.5 Dendrimer, a total of 4,580 protons were found. This would indicate that there was an average of 9-10 NADH-NH_2 molecules per dendrimer. The theoretical number of NADH molecules (16) does not match the calculated values from the NMR spectrum. This could possibly be due to some of the terminal groups not being accessible for conjugation, therefore rendering the reaction less efficient. (Full spectrum can be found in Supplementary Fig. 42)

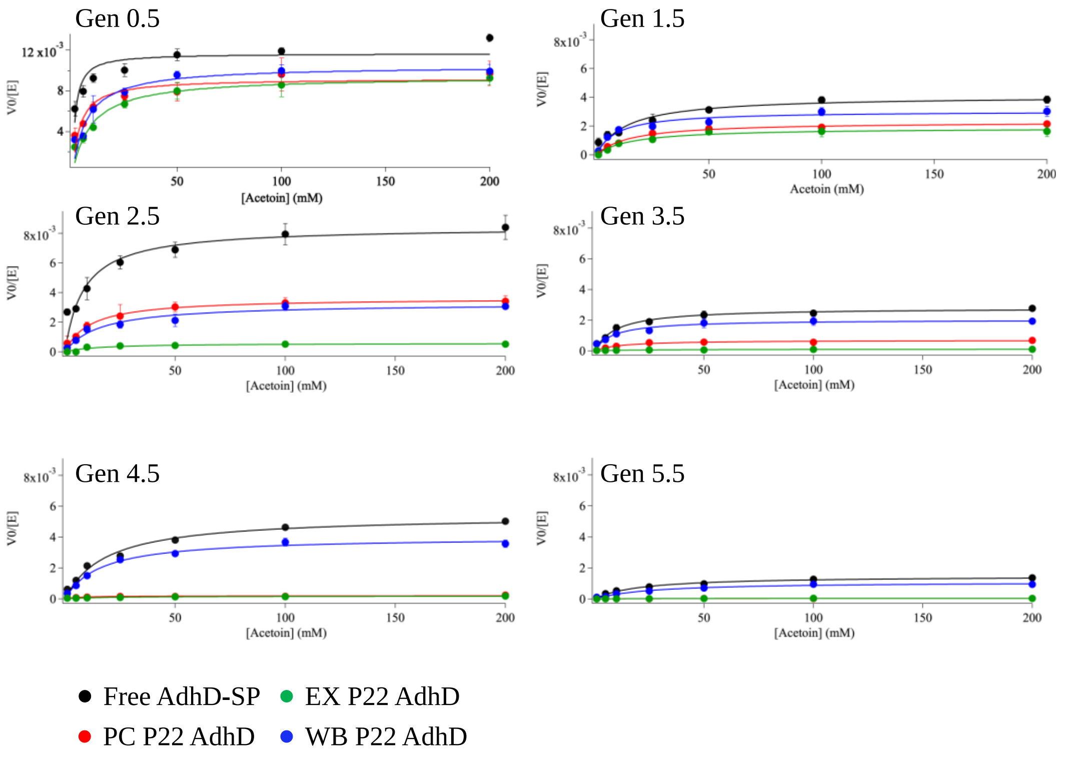

Supplementary Fig.17
Michaelis Menten plots for kinetic experiments completed using negatively charged NADH-Neg$_{xx}$ conjugates in pH 7.0 50 mM Sodium Phosphate100 mM Sodium Chloride Buffer and free AdhD-SP, PC, EX, WB. Error bars = Standard Deviation (n=3).

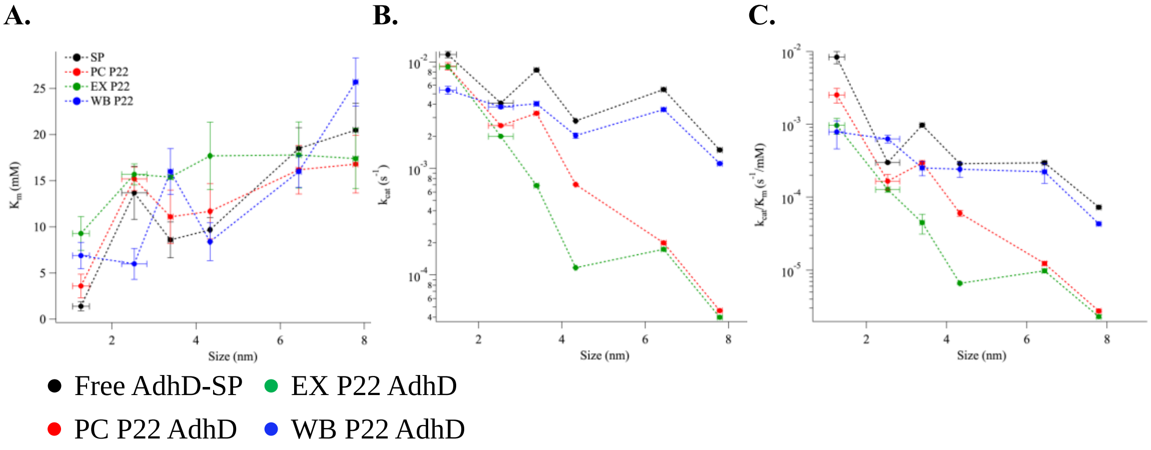

Supplementary Fig.18
The extracted K_M (A), k_{cat} (B),and efficiency (C) values for all NADH-Neg_{xx} conjugates. Horizontal errors bars (n=3) are the standard deviation values for the hydrodynamic radii. Vertical errors bars (n=3) reflect the standard deviation for the turnover values (k_{cat}).

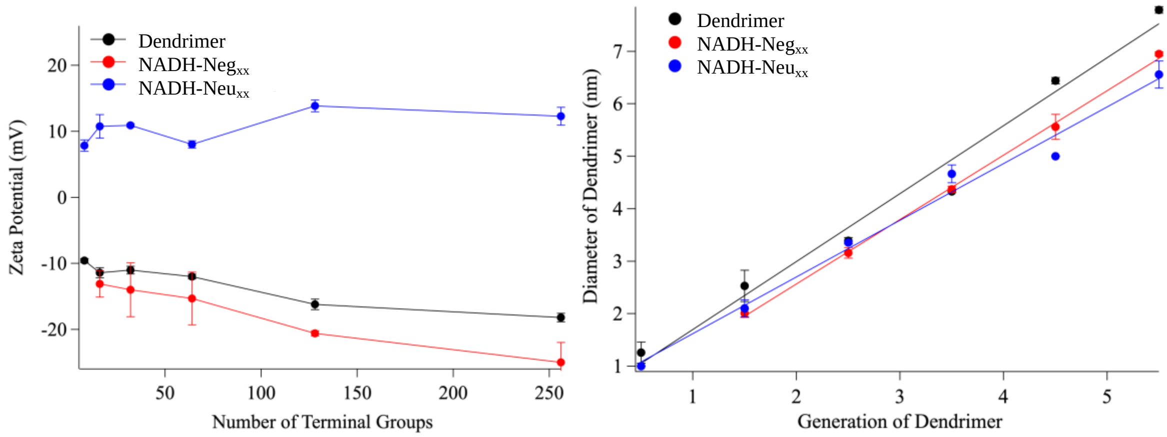

Supplementary Fig.19

The Zeta Potential (A) and DLS (B) values comparing free Dendrimer, NADH-Neg$_{xx}$, and NADH-Neu$_{xx}$. Error bars = Standard Deviation (n=3).

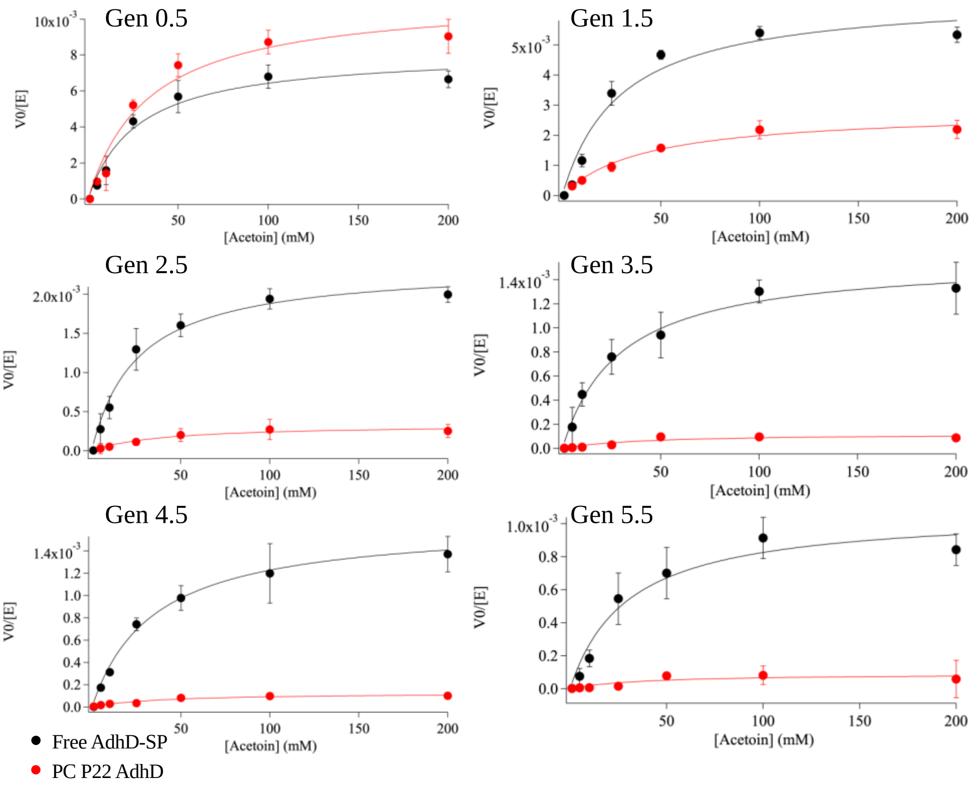

Supplementary Fig.20
Michaelis Menten plots for kinetic experiments completed using neutrally charged NADH-Neu$_{xx}$ conjugates in pH 7.0 50 mM Sodium Phosphate 100 mM Sodium Chloride Buffer and free AdhD-SP and PC. Error bars = Standard Deviation (n=3).

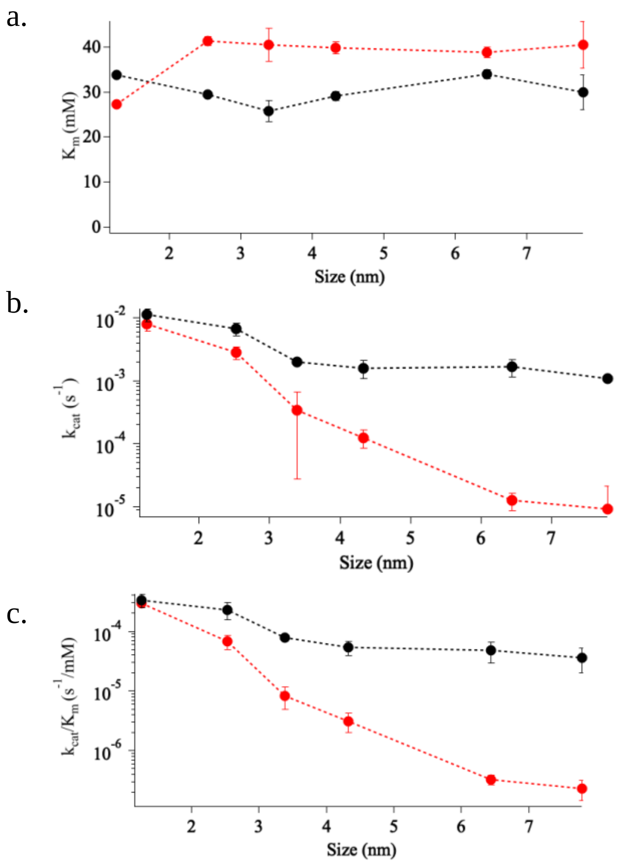

● Free AdhD-SP

● PC P22 AdhD

Supplementary Fig.21

The extracted K_M (A), k_{cat} (B), and efficiency (C) values for all NADH-Neu_{xx} conjugates. Error bars = Standard Deviation (n=3).

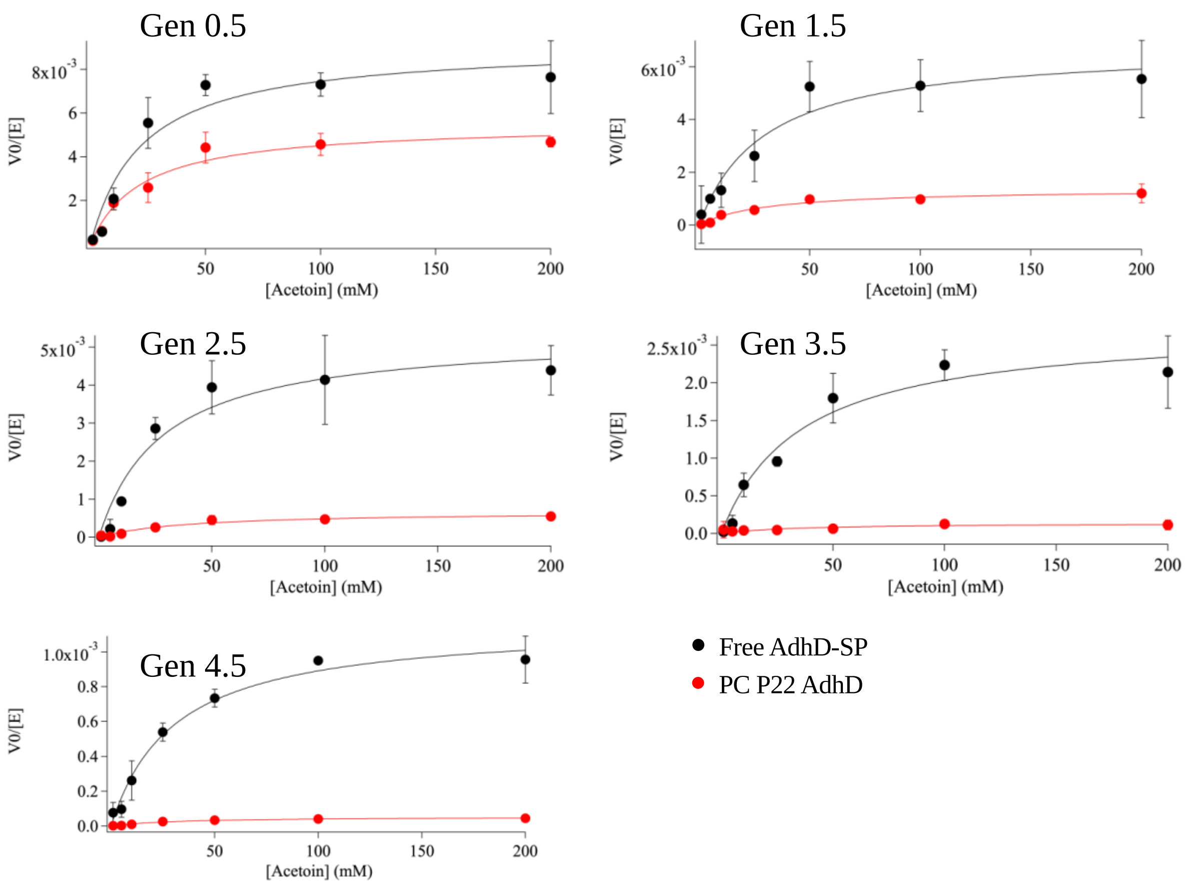

Supplementary Fig.22

Michaelis Menten plots for kinetic experiments completed using NADH-Neg$_{xx(HS)}$ conjugates in pH 7.0 50 mM Sodium Phosphate 400 mM Sodium Chloride Buffer and free AdhD-SP and PC. Error bars = Standard Deviation (n=3).

a.

b.

c.

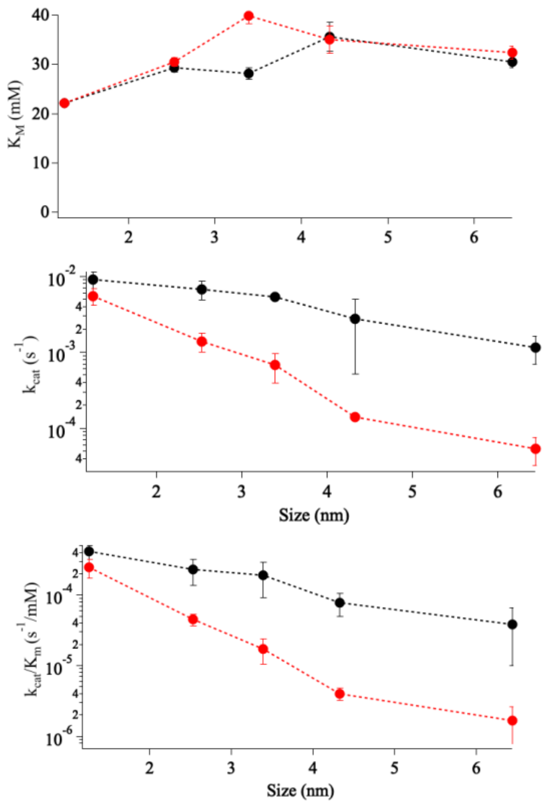

● Free AdhD-SP

● PC P22 AdhD

Supplementary Fig.23

The extracted k_{cat} (A), K_M (B), and efficiency (C) values for all NADH-$Neg_{xx(HS)}$ in pH 7.0 50 mM Sodium Phosphate 400 mM Sodium Chloride Buffer. Error bars = Standard Deviation (n=3).

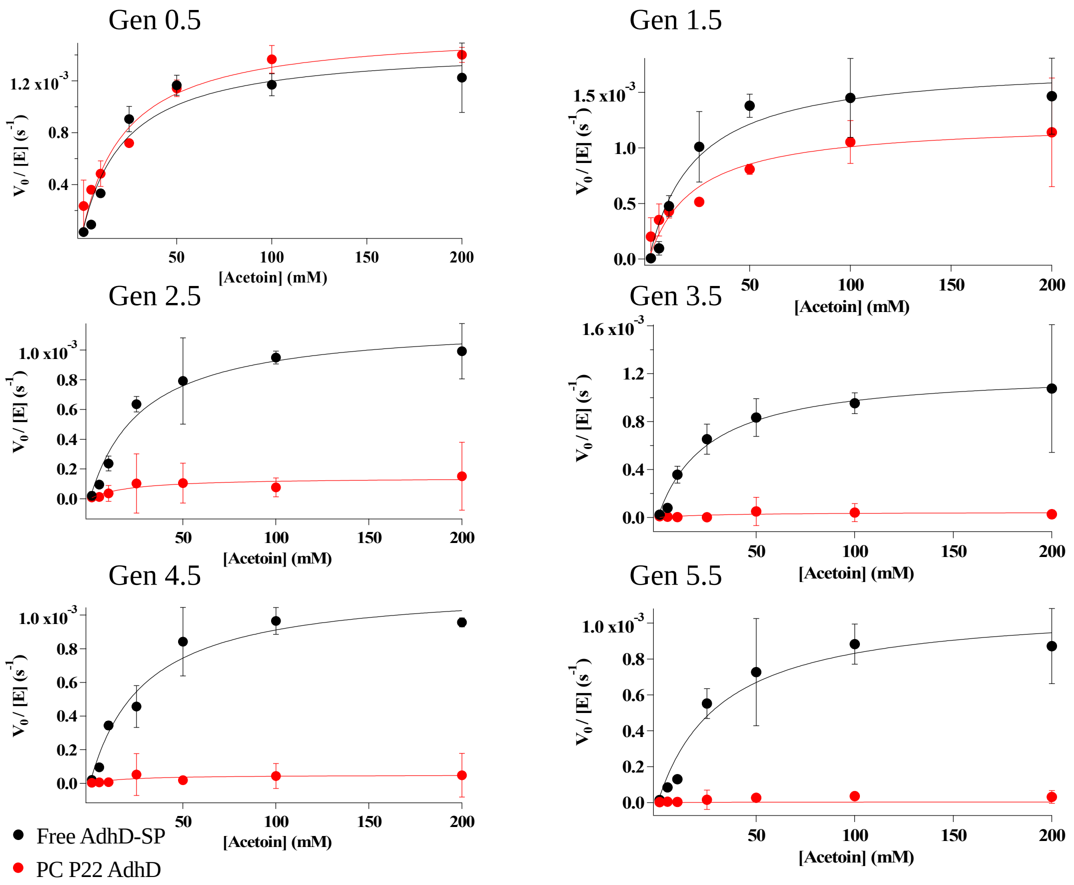

Supplementary Fig.24
Michaelis Menten plots for kinetic experiments completed using NADH-Neu$_{xx(HS)}$ conjugates in pH 7.0 50 mM Sodium Phosphate 400 mM Sodium Chloride Buffer and free AdhD-SP and PC. Error bars = Standard Deviation (n=3).

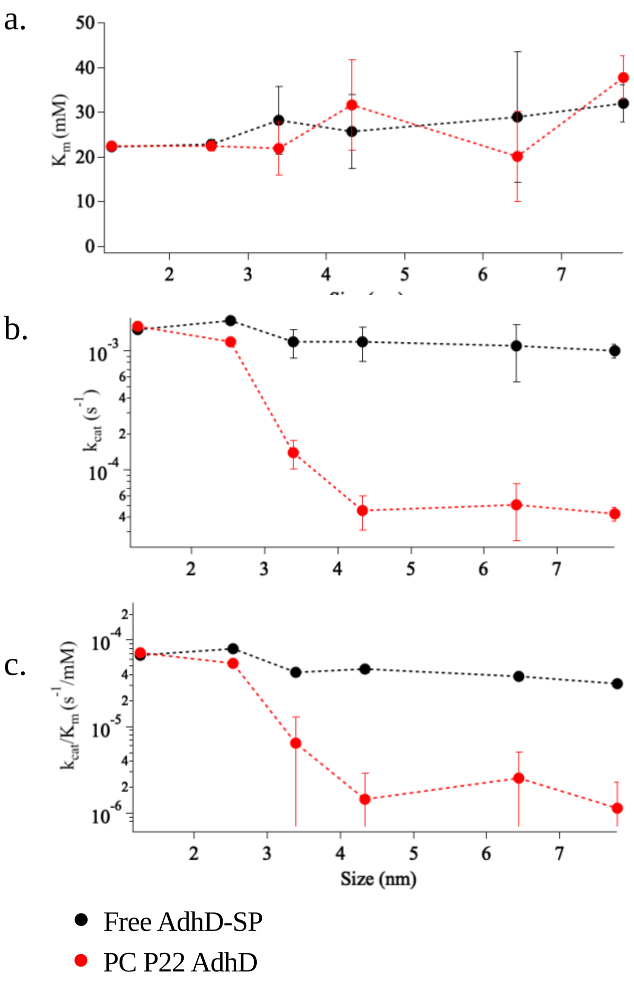

Supplementary Fig.25
The extracted K_M (A), k_{cat} (B),and efficiency (C) values for all NADH-$Neu_{xx(HS)}$ conjugates in pH 7.0 50 mM Sodium Phosphate 400 mM Sodium Chloride Buffer. Error bars = Standard Deviation (n=3).

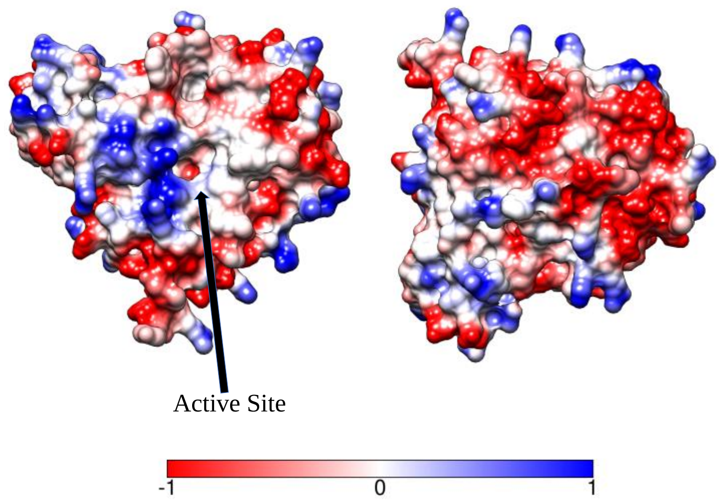

Supplementary Fig.26
Alcohol Dehydrogenase-D coulombic surface was modeled using Chimera Software and settings adjusted to a dielectric constant of 80. The potential ranges from -1 (red) to 1(blue) kcal/mol*e.

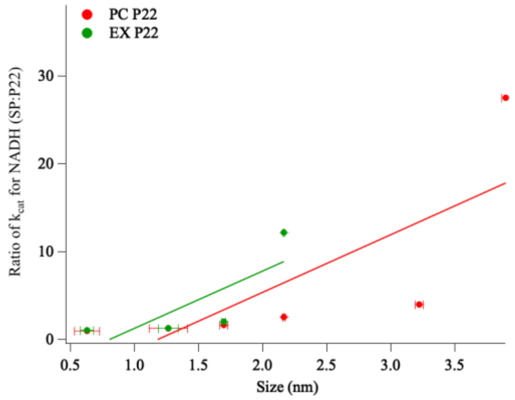

Supplementary Fig.27
The theoretical trend that the activity ratios would follow if P22 particles contained a soft barrier. The dynamic fluctuations in pore sizes would result in a linear trend, rather than ones that illustrated a clear cut-off. Error bars = Standard Deviation (n=3).

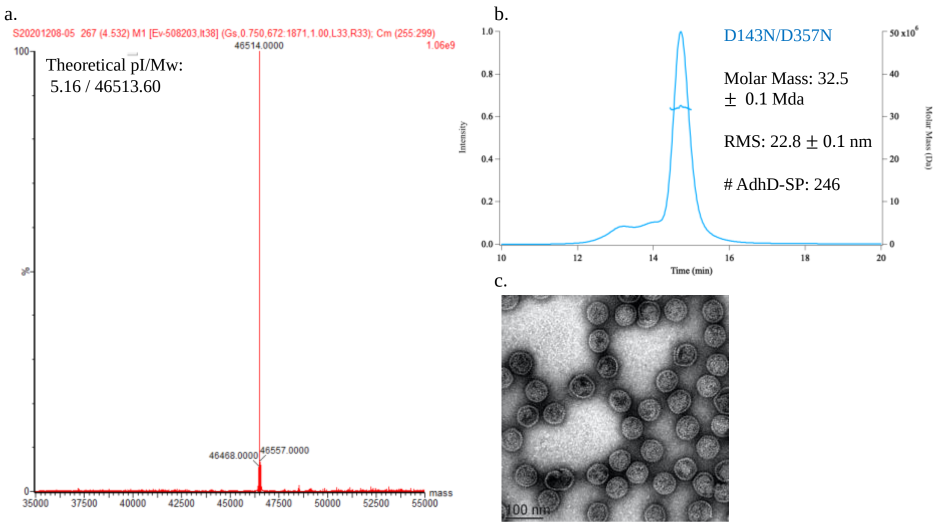

Supplementary Fig.28

Characterization of D143N/D357N P22 Mutant: a) Mass spectroscopy spectrum of the new CP molecular weight matches the theoretical molecular weight well, with minimum other products detected. b) SEC-MALS analysis of the same mutants with the calculated molecular mass, RMS radius, and the number of AdhD-SP cargo molecules encapsulated inside after assembly. c) TEM image of the mutants showing expected size and morphology of the new P22 particles. These are representative images and chromatograms of at least 3 independent experiments each. The TEM images are representative of at least 20 other micrographs.

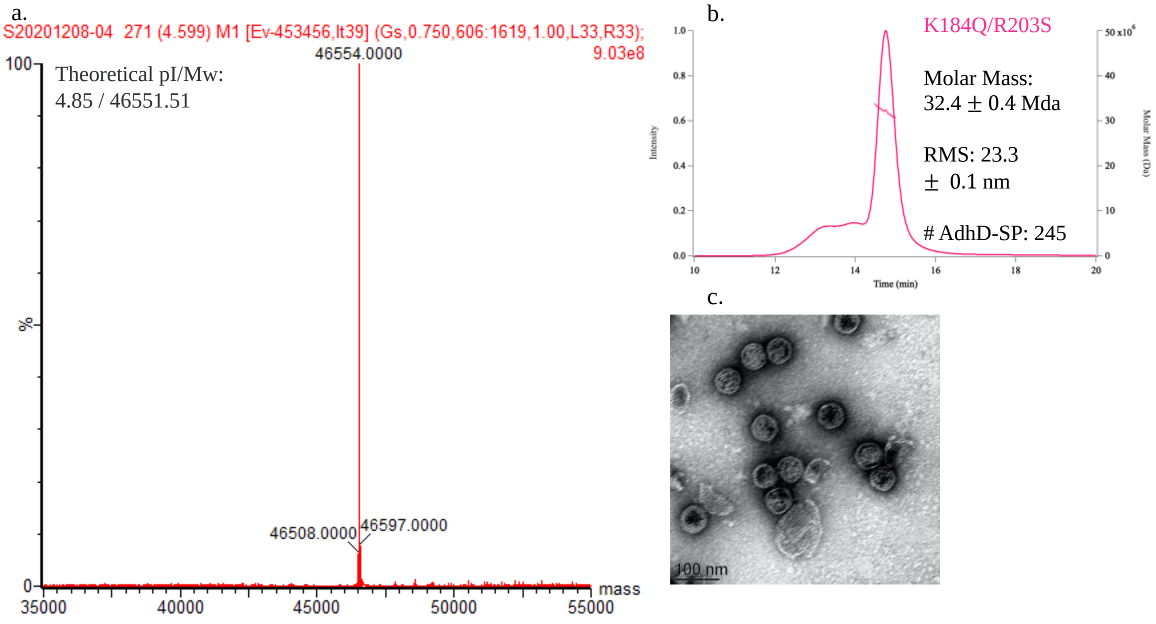

Supplementary Fig.29

Characterization of K184Q/R203S P22 Mutant: a) Mass spectroscopy spectrum of the new CP molecular weight matches the theoretical molecular weight well, with minimum other products detected. b) SEC-MALS analysis of the same mutants with the calculated molecular mass, RMS radius, and the number of AdhD-SP cargo molecules encapsulated inside after assembly. c) TEM image of the mutants showing expected size and morphology of the new P22 particles. These are representative images and chromatograms of at least 3 independent experiments each. The TEM images are representative of at least 20 other micrographs.

a.

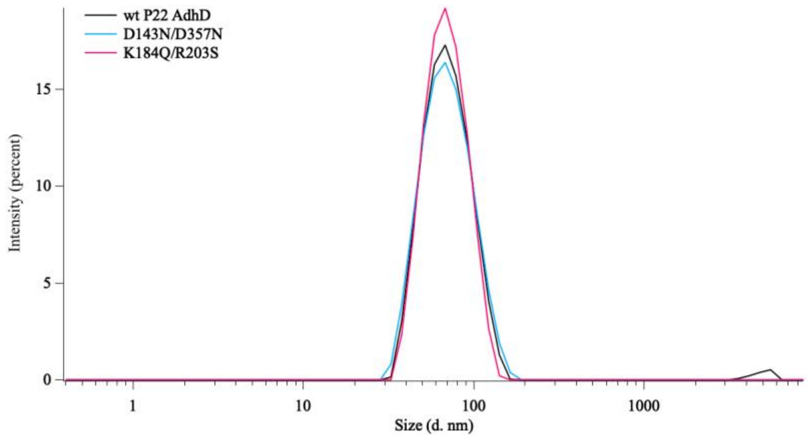

b.

Mutant	*Hydrodynamic Radius(nm)*	*std. dev. (+/-)*	*Mutant*	*Zeta Potential (mV)*	*std. dev. (+/-)*
**wt P22 AdhD**	**25.7**	**0.2**	**wt P22 AdhD**	**-23.8**	**1.2**
**D134N/D357N**	**26.1**	**0.6**	**D134N/D357N**	**-19.8**	**0.7**
**K184Q/R203S**	**25.0**	**0.4**	**K184Q/R203S**	**-25.2**	**1.1**

Supplementary Fig.30
Dynamic Light Scattering (DLS) and Zeta Potential measurements collected for K184Q/R203S and D143N/D357N P22 Mutants: a) DLS data comparing the two mutants to wtP22 particles (all three contain the same AdhD-SP cargo encapsulated) b) The measured hydrodynamic radius and zeta potential for the 2 mutants and wtP22. Error bars = Standard Deviation (n=3).

a.

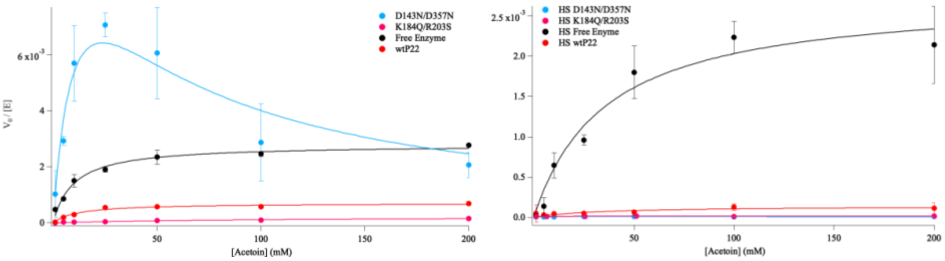

b.

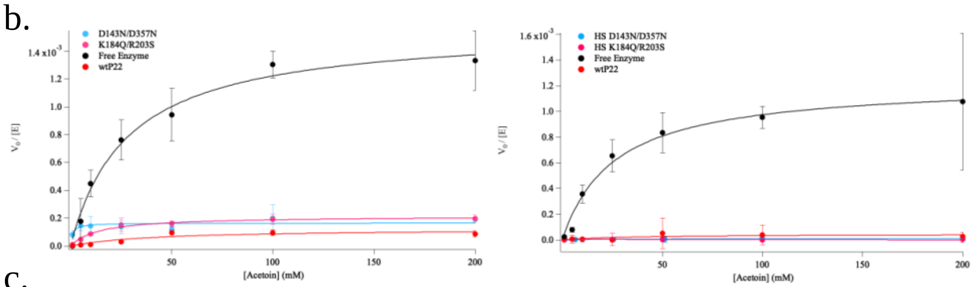

c.

**Km**	**High Salt**
D143N/D357N + NADH-Neg$_{3.5}$ = 5.8 mM	= 1.2 mM
D143N/D357N + NADH-Nue$_{3.5}$= 1.3 mM	= 1.2 mM
K184Q/R203S + NADH-Neg$_{3.5}$= 92.5 mM	= 1.2 mM
K184Q/R203S + NADH-Neu$_{3.5}$= 14.6 mM	= 5.0 mM

**k_{cat}**	**High Salt**
D143N/D357N + NADH-Neg$_{3.5}$ = 0.013 s^{-1}	= 0.00034 s^{-1}
D143N/D357N + NADH-Nue$_{3.5}$= 0.00017 s^{-1}	= 0.000050 s^{-1}
K184Q/R203S + NADH-Neg$_{3.5}$= 0.00022 s^{-1}	= 0.00038 s^{-1}
K184Q/R203S + NADH-Neu$_{3.5}$= 0.00022 s^{-1}	= 0.000045 s^{-1}

Supplementary Fig.31
Michaelis Menten kinetics data collected using the two new mutants (D143N/D357N and K184Q/R203S), wtP22, and free enzyme (AdhD-SP: a/b) Experiments completed using (a)NADH-Neg$_{3.5}$ and (b)NADH-Neu$_{3.5}$ conjugates in pH 7.0 50 mM Sodium Phosphate (left) and 400 mM Sodium Chloride Buffer (right) compared to free AdhD-SP and wtP22. All data followed a nonlinear regression curve except for in the case of mutant D143N/D357N with NADH-Neg$_{3.5}$ (top left), which was fit using a nonlinear regression with inhibition. c) Extracted K_m and k_{cat} values for all the conditions used with the P22 mutant particles. Error bars = Standard Deviation (n=3).

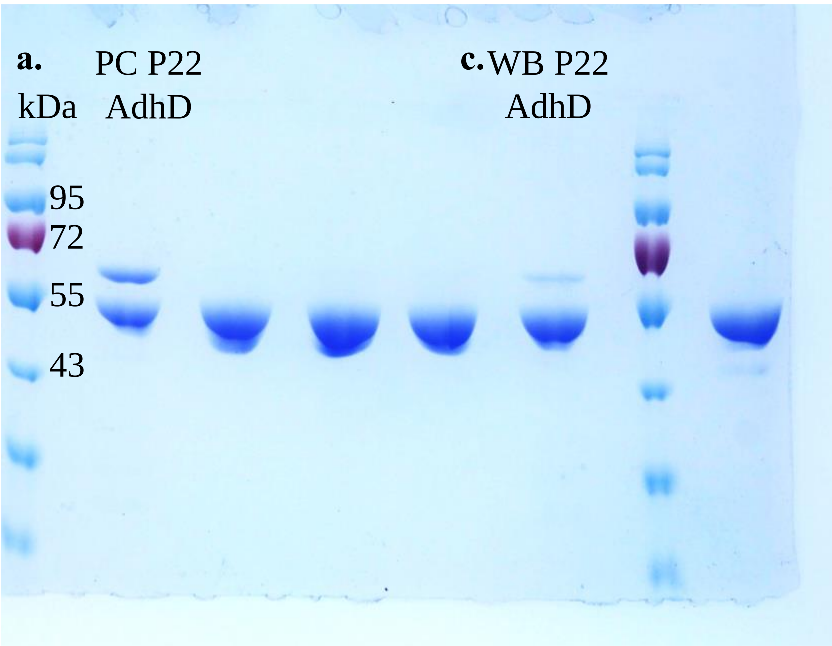

Supplementary Fig.32
Full SDS gel croppe0.000d in Figure 1a and 1c for PC and WB P22 particles. All other lanes show P22 CP and are not relevant to the work here.

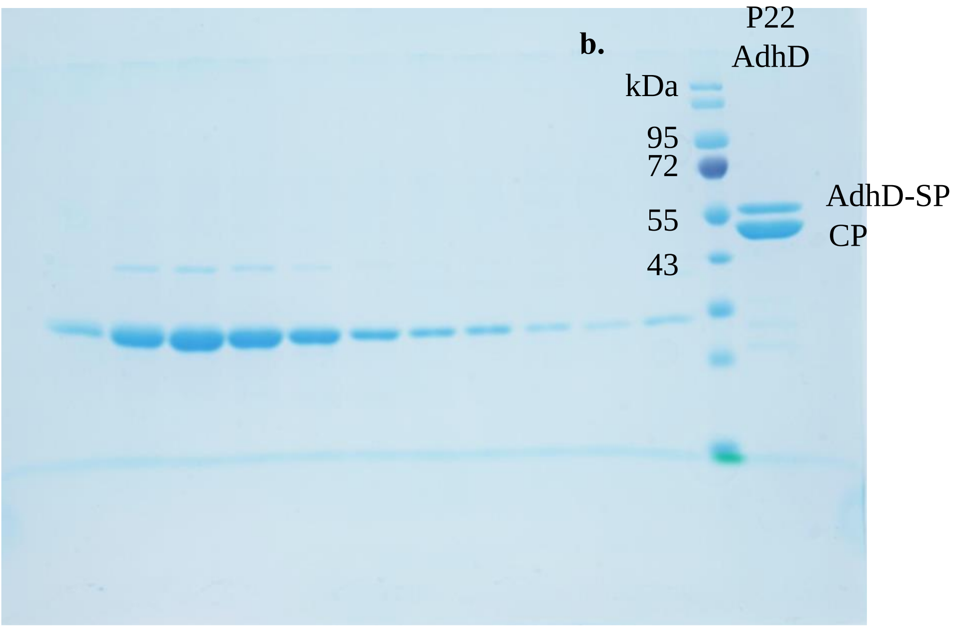

Supplementary Fig.33
Full SDS gel cropped in Figure 1b for EX particles. All other lanes are not relevant to the work here.

Full NMR Spectra

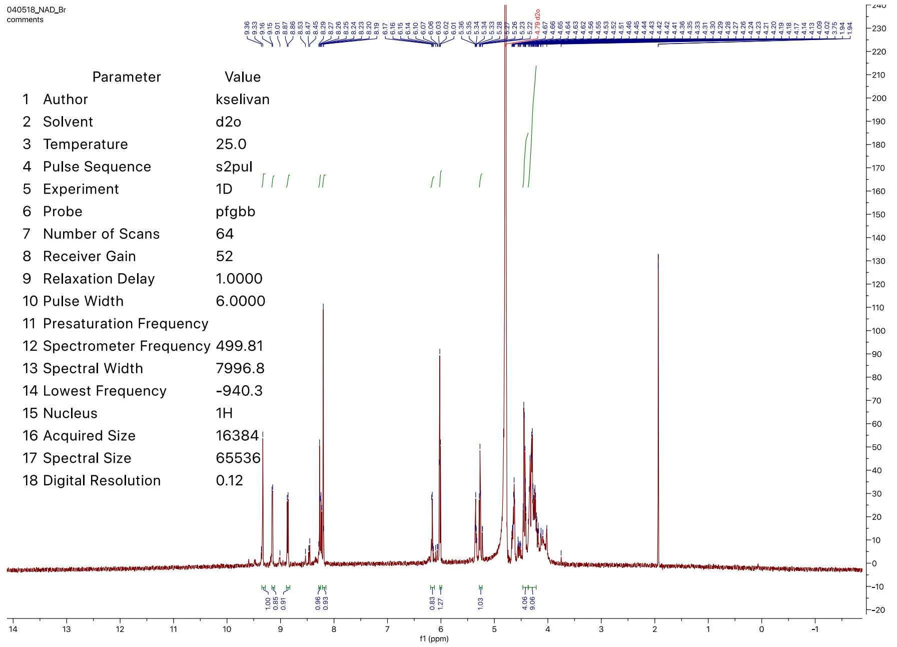

Supplementary Fig.34: NAD^{+}-Br

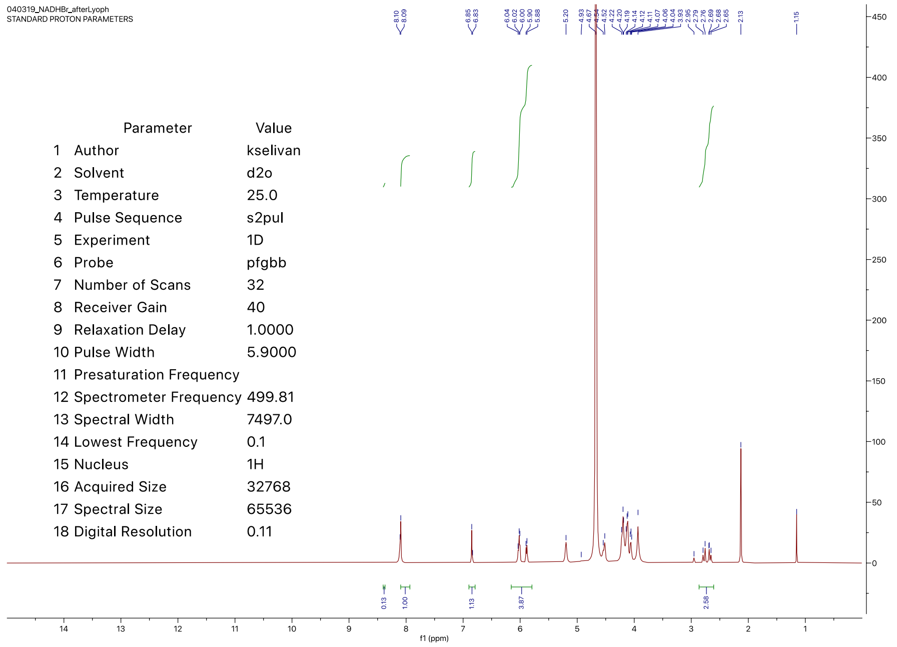

Supplementary Fig.35: NADH-Br

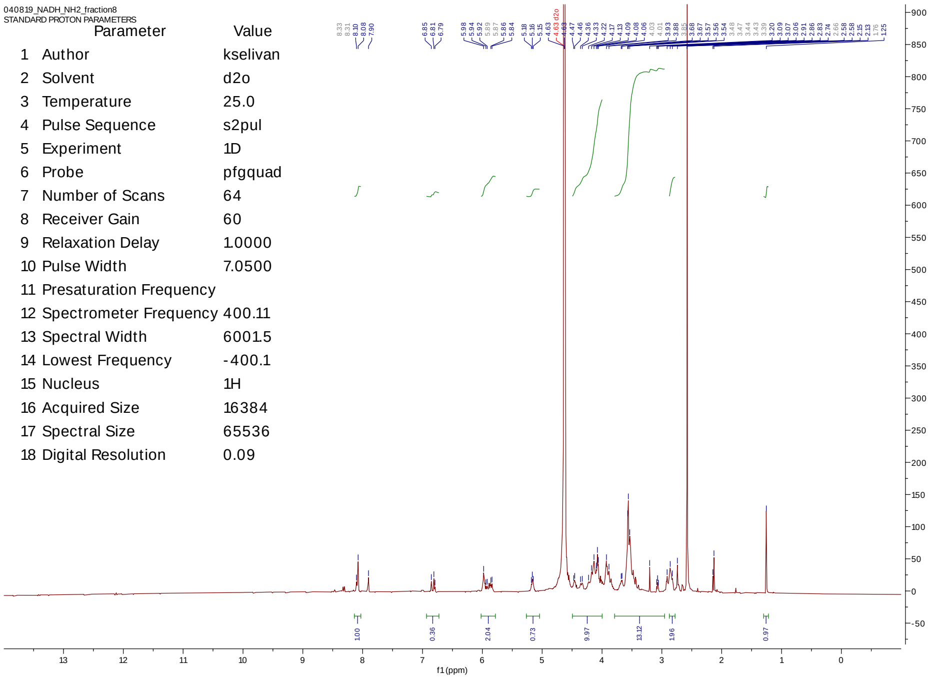

Supplementary Fig.36: NADH-NH_2

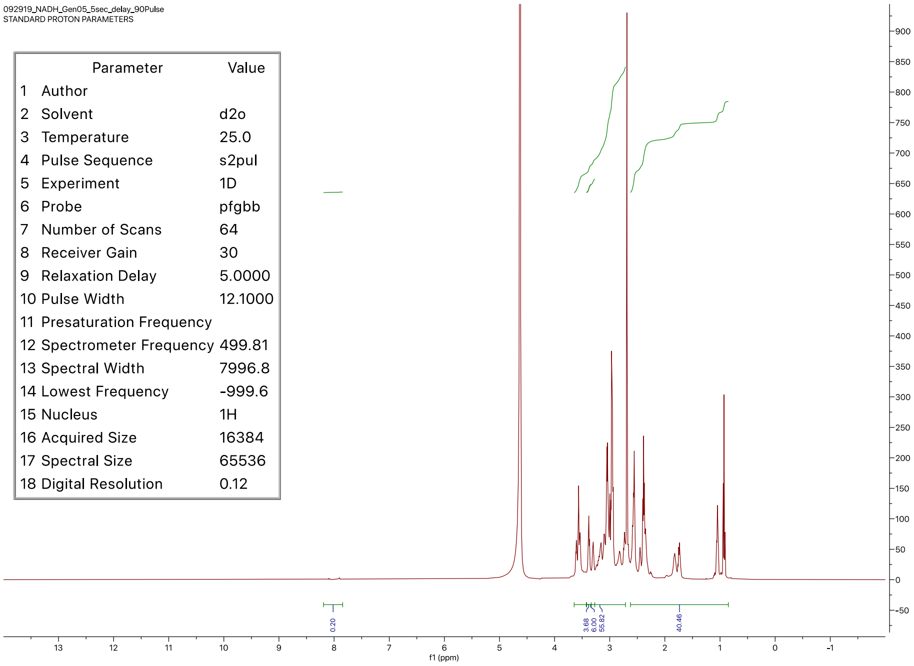

Supplementary Fig.37: NADH-$Neg_{0.5}$

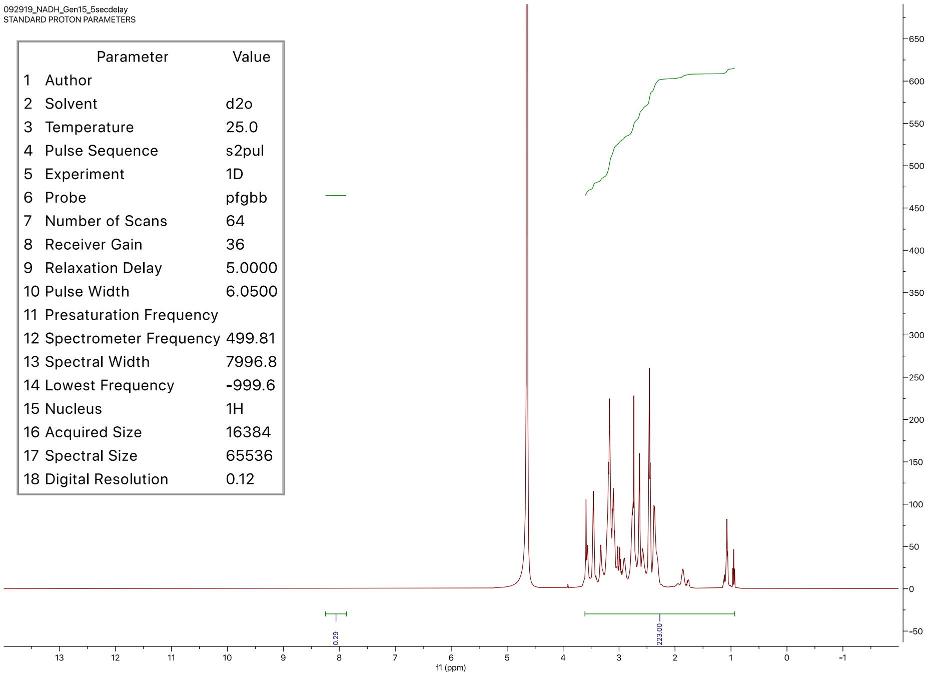

Supplementary Fig.38: NADH-Neg$_{1.5}$

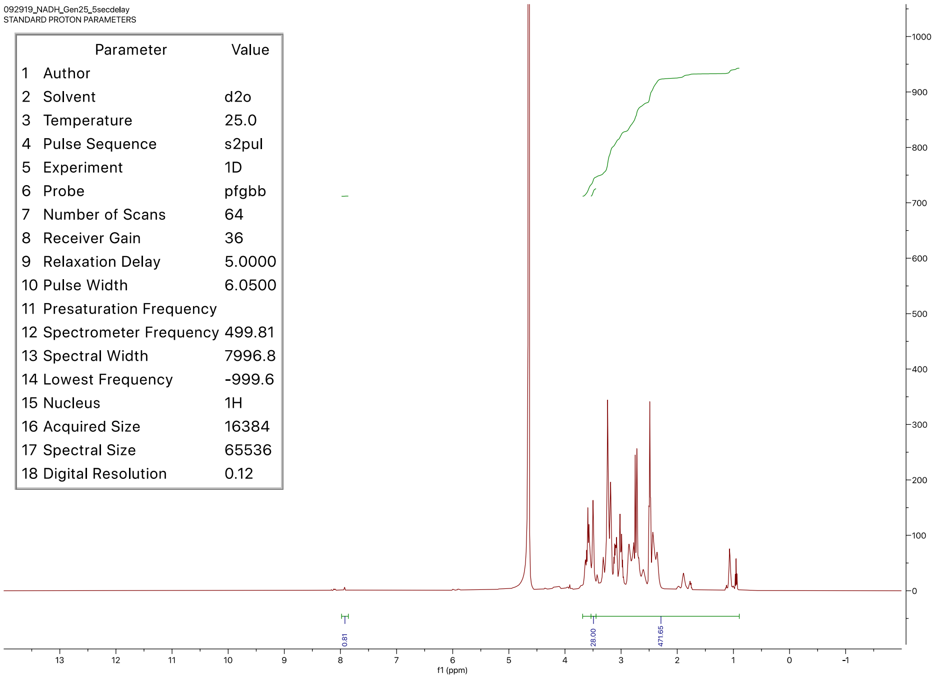

Supplementary Fig.39: NADH-Neg$_{2.5}$

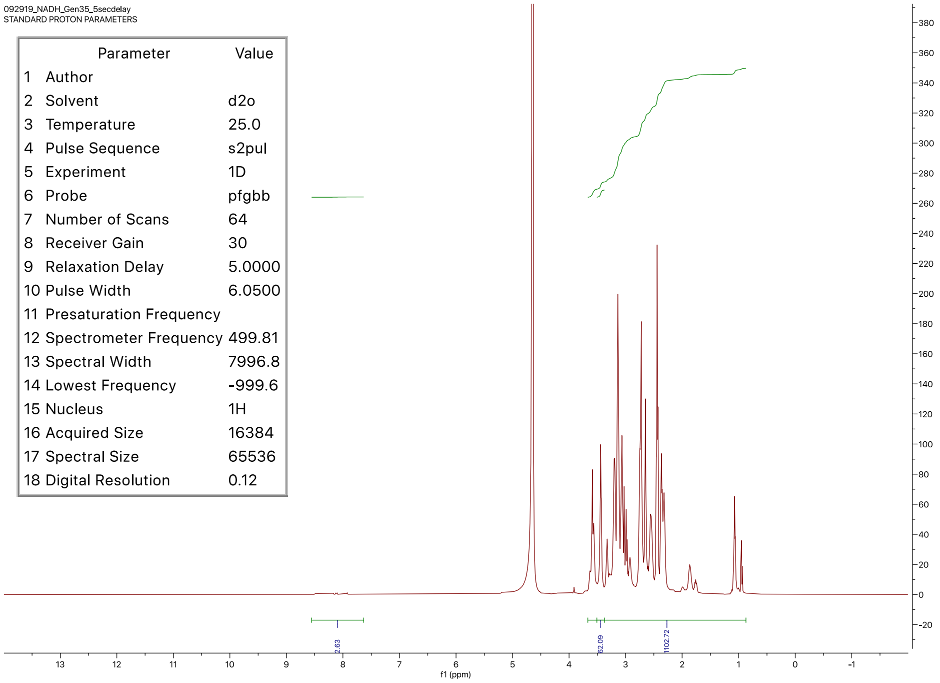

Supplementary Fig.40: NADH-Neg$_{3.5}$

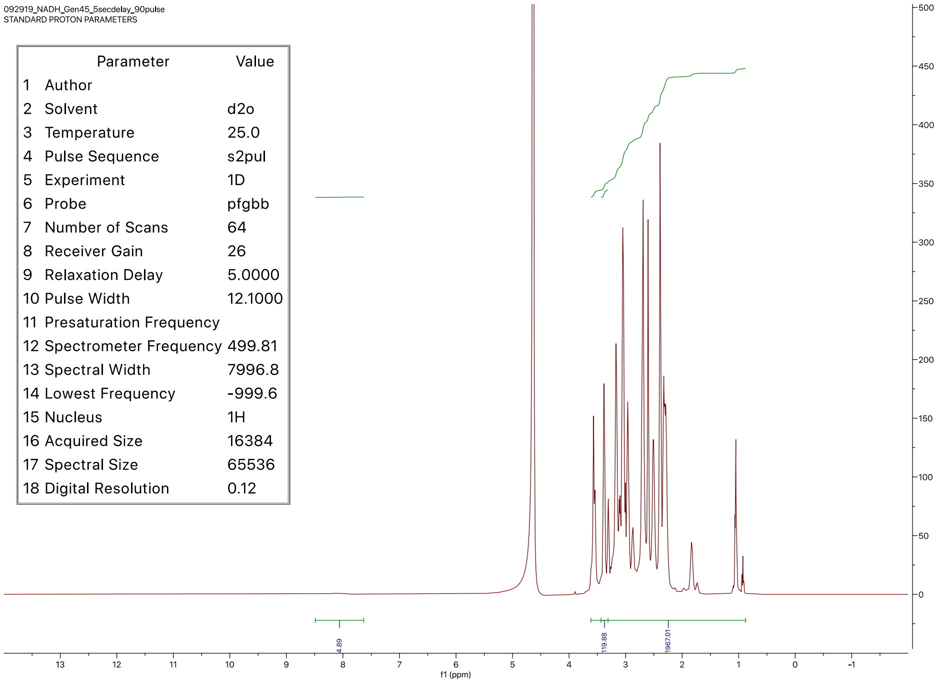

Supplementary Fig.41: NADH-Neg$_{4.5}$

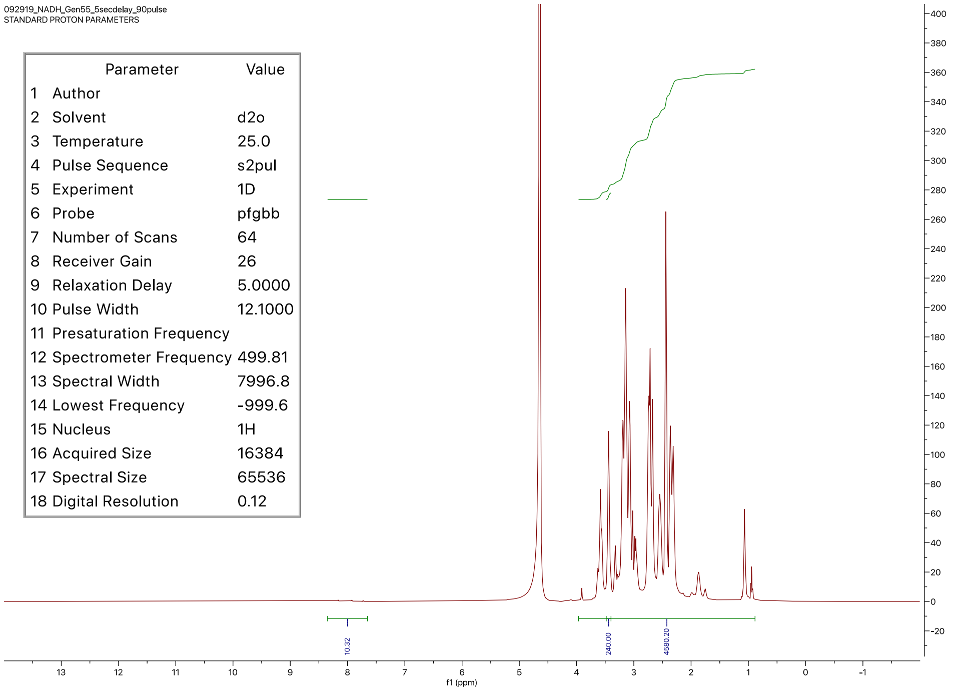

Supplementary Fig.42: NADH-Neg5.5
