# Supplementary material for: Molecular exclusion limits for diffusion across a porous capsid
Source: Nat Commun. 2021 May 18;12:2903. doi: 10.1038/s41467-021-23200-1 (PMC8131759; doi:10.1038/s41467-021-23200-1)
Supplement: Supplementary file 2 — Description of Additional Supplementary Files [file 41467_2021_23200_MOESM2_ESM.docx]

**Description of Additional Supplementary Files**

**Supplementary Movie 1:**

360° View of Electrostatic Potential Map of the PC P22 Asymmetric Unit
